# Inter-individual variation in chlorpyrifos toxicokinetics characterized by physiologically based kinetic (PBK) and Monte Carlo simulation comparing human liver microsome and Supersome^™^ cytochromes P450 (CYP)-specific kinetic data as model input

**DOI:** 10.1007/s00204-022-03251-z

**Published:** 2022-03-16

**Authors:** Shensheng Zhao, Sebastiaan Wesseling, Ivonne. M. C. M. Rietjens, Marije Strikwold

**Affiliations:** 1 Division of Toxicology, Wageningen University and Research, Stippeneng 4, 6708 WE Wageningen, The Netherlands; 2 Van Hall Larenstein University of Applied Sciences, 8901 BV Leeuwarden, The Netherlands

**Keywords:** Chlorpyrifos (CPF), Red blood cell (RBC) acetylcholinesterase (AChE) inhibition, Inter-individual differences, Physiologically based kinetic (PBK) modeling, Monte Carlo (MC) simulation

## Abstract

The present study compares two approaches to evaluate the effects of inter-individual differences in the biotransformation of chlorpyrifos (CPF) on the sensitivity towards in vivo red blood cell (RBC) acetylcholinesterase (AChE) inhibition and to calculate a chemical-specific adjustment factor (CSAF) to account for inter-individual differences in kinetics (HK_AF_). These approaches included use of a Supersome^™^ cytochromes P450 (CYP)-based and a human liver microsome (HLM)-based physiologically based kinetic (PBK) model, both combined with Monte Carlo simulations. The results revealed that bioactivation of CPF exhibits biphasic kinetics caused by distinct differences in the Km of CYPs involved, which was elucidated by Supersome^™^ CYP rather than by HLM. Use of Supersome^™^ CYP-derived kinetic data was influenced by the accuracy of the intersystem extrapolation factors (ISEFs) required to scale CYP isoform activity of Supersome^™^ to HLMs. The predicted dose–response curves for average, 99th percentile and 1st percentile sensitive individuals were found to be similar in the two approaches when biphasic kinetics was included in the HLM-based approach, resulting in similar benchmark dose lower confidence limits for 10% inhibition (BMDL_10_) and HK_AF_ values. The variation in metabolism-related kinetic parameters resulted in HK_AF_ values at the 99th percentile that were slightly higher than the default uncertainty factor of 3.16. While HK_AF_ values up to 6.9 were obtained when including also the variability in other influential PBK model parameters. It is concluded that the Supersome^™^ CYP-based approach appeared most adequate for identifying inter-individual variation in biotransformation of CPF and its resulting RBC AChE inhibition.

**Supplementary Information:**

The online version contains supplementary material available at 10.1007/s00204-022-03251-z.

## Introduction

Chlorpyrifos (CPF) is the organophosphate most studied in the past decades due to its intensive use as an insecticide in nearly 100 countries (The Dow Chemical Company). Inhibition of acetylcholinesterase (AChE) following irreversible binding of the potent metabolite of CPF chlorpyrifos-oxon (CPO), has been characterized as the main cause of acute CPF exposure-related (neuro)toxicity (Satoh and Gupta 2011). On 10 January 2020, The European Commission formally adopted regulations that revoke the renewal of approval for CPF (European Commision 2020), because: (i) the potential genotoxicity of CPF remained unclear, leading the European Food Safety Authority (EFSA) to conclude that no toxicological reference dose could be derived, hampering the risk assessment for consumers, operators, workers, bystanders and residents; (ii) developmental neurotoxicity has been observed in epidemiological studies; and iii) CPF is classified as reproduction category 1B (regarding developmental toxicity) (EFSA 2019). Recently, although more and more countries ban the use of CPF or allow its use only under certain restrictions, CPF residues are still frequently and widely found in food, and in some samples, residue levels have been reported to exceed the European Union Maximum Residue Levels (EUMRL) or to result in exposures above the acute reference dose (ARfD) (EFSA 2020; HEAL and PAN Europe 2019; Hongsibsong et al. 2020). Given that measuring the AChE inhibition in the nervous system is not straightforward, measurement of red blood cell (RBC) AChE inhibition has been widely used as a surrogate endpoint to derive points of departure (PODs) in human risk assessment for organophosphate pesticides (OPs) including CPF (EFSA 2014; USEPA 2014). Also in the present study, RBC AChE inhibition was used as critical adverse effect for the assessment.

**Fig. 1:**
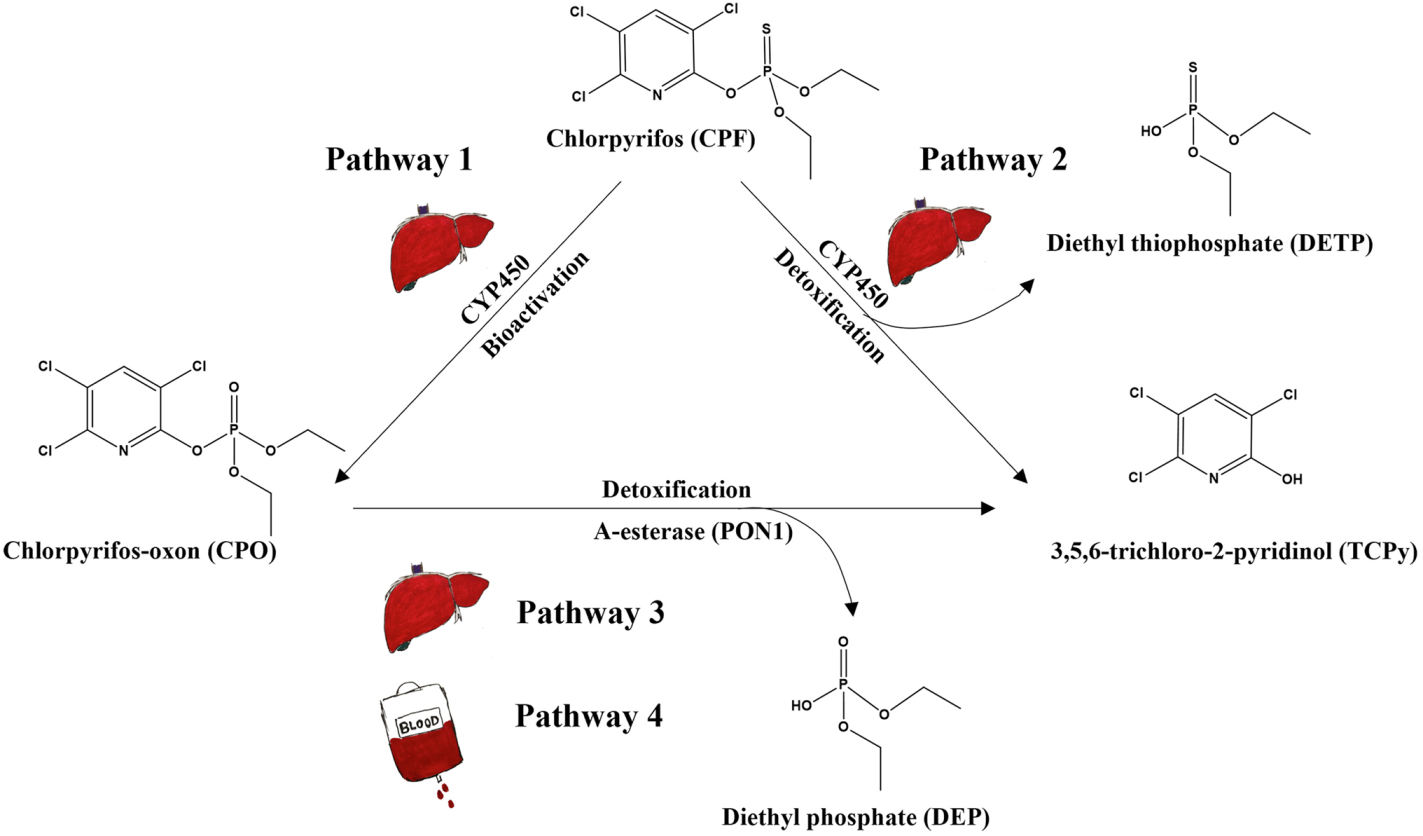
Proposed metabolic pathways of chlorpyrifos in human

The metabolic pathways of CPF (Fig. 1) have been well characterized and include: (i) bioactivation of CPF to the potent AChE inhibitor CPO by cytochromes P450 (CYPs) (pathway 1) (Foxenberg et al. 2007; Sams et al. 2004); (ii) detoxification of CPF to 3,5,6-trichloro-2-pyridinol (TCPy) and diethyl thiophosphate (DETP) by CYPs (pathway 2) (Foxenberg et al. 2007; Sams et al. 2004); (iii) detoxification of the bioactive metabolite CPO to TCPy and diethyl phosphate (DEP) by paraoxonase1 (PON1) present in liver (pathway 3) and in plasma (pathway 4) (Furlong et al. 1989; Timchalk et al. 2002). Available data (Foxenberg et al. 2007) indicate that the bioactivation reaction is mainly mediated by the isoforms CYP2B6, CYP1A2 and to a lesser extent by CYP2C19, CYP3A4, CYP3A5 and CYP3A7, while the detoxification reaction is mainly catalyzed by CYP2C19 and CYP1A2, and to a lesser extent by CYP2B6, CYP3A4 as well as CYP3A5. Additionally, Foxenberg et al. (2007) also pointed out that the Michaelis–Menten constant (*K*_m_) of CYP1A2, CYP2B6, CYP2C19 towards CPF is one to two orders of magnitude lower than that of CYP3A4, indicating the possible primary roles of CYP1A2, CYP2B6, CYP2C19 in CPF biotransformation at low level exposure.

Previous studies have shown that the expression and activity of enzymes involved in CPF biotransformation are highly variable among the general human population (Lang et al. 2001; Tracy et al. 2016; Westlind-Johnsson et al. 2003). For example, a 100-fold difference in protein levels of CYP2B6 between human individuals (Lang et al. 2001), a 40-fold inter-individual variation in protein levels of CYP3A4 (Westlind-Johnsson et al. 2003) and up to 20-fold variation in CYP2C19 expression within different individuals (Ellison et al. 2012; Koukouritaki et al. 2004) have been shown. Moreover, PON1 shows a 40-fold inter-individual variation in activity (Coasta et al. 2006). Such inter-individual variability in the expression of CPF biotransformation-related enzymes may ultimately lead to inter-individual differences in sensitivity toward CPF poisoning, because the susceptibility of human individuals towards CPF poisoning is determined by the balance between bioactivation of CPF and detoxification of CPF and CPO in the human body (Poet et al. 2003; Timchalk et al. 2002).

To address inter-individual human variation in metabolism upon exposure to different compounds, so-called physiologically based kinetic (PBK) modeling has been used. To apply this approach for CPF, the related metabolic parameters and their inter-individual variability must be quantified. This can be done using either Supersome^™^ CYPs (from now on written as Supersome^™^, genetically engineered enzyme systems with a relatively high catalytic activity of an individual CYP) or human liver microsomes (HLMs). In the present study, both methods were applied to allow a comparison. The kinetic parameters derived by either Supersome^™^ or HLM in vitro were used as input for the PBK model to further assess the inter-individual variation in metabolism of CPF and the resulting susceptibility towards CPF exposure-induced RBC AChE inhibition. Theoretically, the HLM-based PBK model approach can capture and represent variation in hepatic CYP-mediated metabolism in different human individuals well, when variation in HLMs is described based on kinetic data obtained from individual HLMs. However, this approach normally requires large number of individual HLMs to capture the variation representative for the population as a whole, and the obtained kinetic data cannot be used as a basis for extrapolation when deriving the distribution of other age, gender and ethnicity groups (Foxenberg et al. 2007; Knaak et al. 2004). To circumvent these limitations, the Supersome^™^-based PBK approach can be used, as it is based on the combination of the intrinsic activity of CYP isoforms for the compound of interest and intersystem extrapolation factors (ISEFs), to scale CYP isoform activities in the Supersome^™^ toward HLMs, and subsequent multiplication with the hepatic abundance of CYPs representative for the target individuals of interest (i.e. with regard to age, gender, ethnicity, etc.) (Proctor et al. 2004). Such an approach thus provides the possibility to extrapolate in vitro-derived metabolic kinetic constants from a set of relevant Supersomes^™^ toward in vivo values for various target groups.

The aim of the present study was to compare the applicability and outcome of the Supersome^™^-based PBK model approach and the HLM-based PBK model approach to characterize inter-individual variability in metabolism of CPF and its resulting RBC AChE inhibition, and calculate a chemical-specific adjustment factor (CSAF) for the inter-individual variation in kinetics (HK_AF_) for CPF. To this end, experiment-derived and literature-obtained kinetic parameters of Supersomes^™^, HLMs and human plasmas (HP) were collected and used as input for the Supersome^™^-based PBK model and HLM-based PBK model, which were both developed based on the previously developed CPF PBK model (Zhao et al. 2019). Furthermore, the two models were extended to include Monte Carlo simulations and coefficients of variation (CVs) for each kinetic parameter, and other influential parameters to predict inter-individual kinetic variation. Finally, PBK model-based reverse dosimetry was applied to predict in vivo dose–response curves for RBC AChE inhibition by translating in vitro recombinant human AChE (rhAChE) inhibition data for the average and sensitive sub-groups within the adult population.

## Materials and methods

### Chemicals and biological materials

CPF, TCPy, acetylthiocholine iodide (ATC), 5,5’-dithiobis (2-nitrobenzoic acid) (DTNB), bovine serum albumin (BSA), tetraisopropyl pyrophosphoramide (iso-OMPA), reduced nicotinamide adenine dinucleotide phosphate (NADPH), sodium phosphate dibasic dihydrate (Na_2_HPO_4_·2H_2_O), sodium phosphate monobasic dihydrate (NaH_2_PO_4_·2H_2_O), phenacetin, acetaminophen, bupropion, ( ±)-hydroxybupropion solution, 4-hydroxymephenytoin, testosterone, 6β-hydroxytestosterone solution, and trizma^®^base were purchased from Sigma-Aldrich (Zwijndrecht, The Netherlands). (S)-Mephenytoin was purchased from Santa Cruz Biotechnology, Inc. (Dallas, TX, USA). CPO was purchased from TRC-Canada (Toronto, Ontario, Canada). Magnesium chloride hexahydrate (MgCl_2_·6H_2_O), hydrochloric acid (HCl), sodium hydroxide (NaOH), ethylenediaminetetraacetic acid disodium salt dihydrate (EDTA), trifluoroacetic acid (TFA), formic acid and calcium chloride dihydrate (CaCl_2_·2H_2_O) were purchased from VWR International (Amsterdam, The Netherlands). Acetonitrile (ACN, UPLC/MS grade) and methanol (UPLC/MS grade) were purchased from Biosolve (Valkenswaard, The Netherlands). Pierce^™^ BCA protein assay kit was purchased from Thermo Fisher Scientific (Rockford, IL, USA).

HLMs (pooled from 150 donors, mixed gender), human Supersomes^™^ (human CYP1A2 + reductase, human CYP2B6 + reductase + cytochrome b5, human CYP2C19 + reductase + cytochrome b5, human CYP3A4 + reductase + cytochrome b5) were purchased from Corning (Amsterdam, The Netherlands). 25 Individual HP samples of Caucasian origin (13 males and 12 females within the age range from 25 to 75 years old) were purchased from BioIVT (West Sussex, UK). rhAChE was purchased from Sigma-Aldrich Co. (St. Louis, MO, USA), and in solutions, the enzyme was stabilized with 1 mg/ml BSA (Kaushik et al. 2007).

### Outline of PBK-based reverse dosimetry approaches with Monte Carlo simulation

**Fig. 2:**
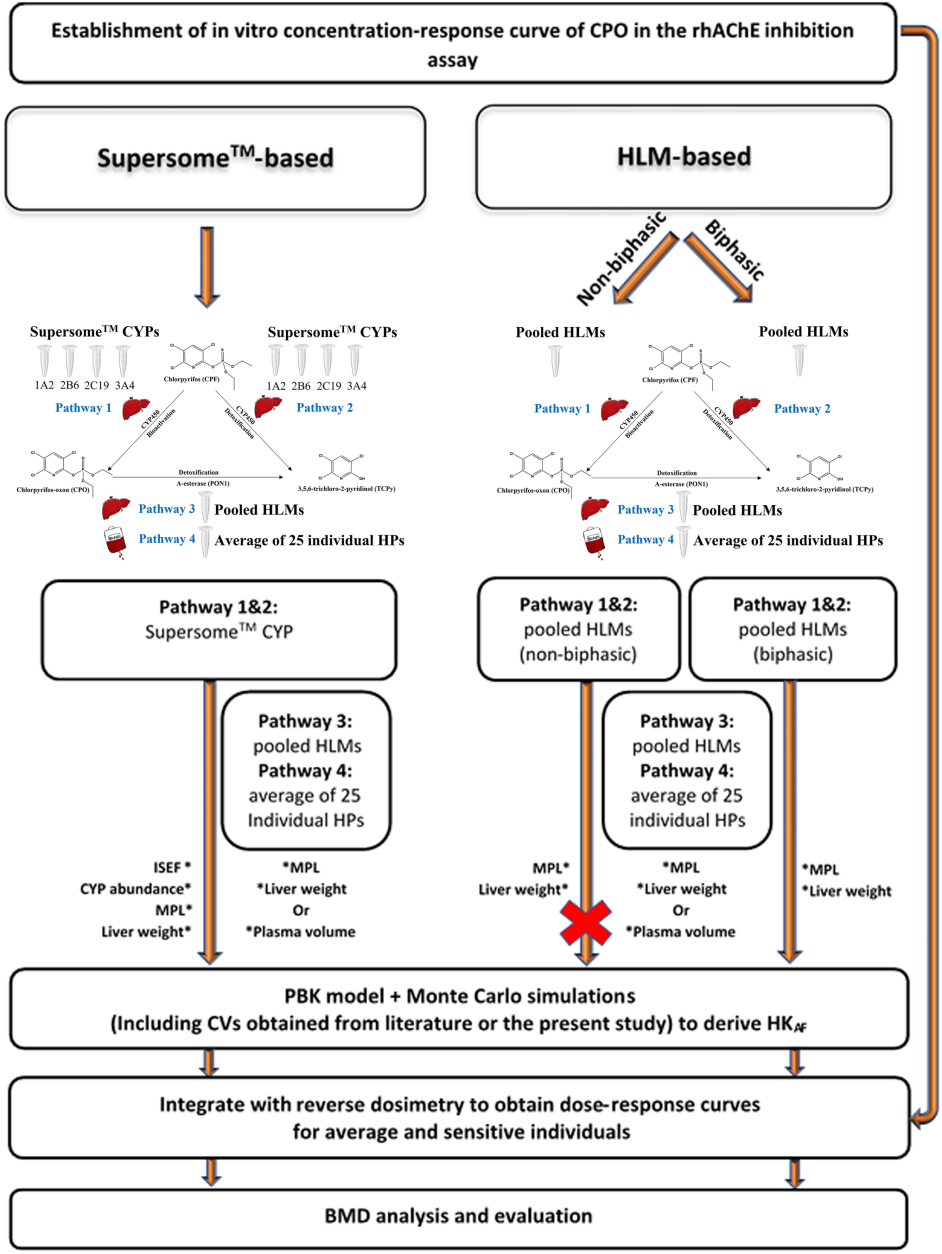
Schematic presentation of the two approaches that were applied in the present study to assess inter-individual variation in the biotransformation of CPF and its resulting HK_AF_ values as well as dose–response curves for CPF-mediated RBC AChE inhibition. CYP is Cytochrome P450, CPF is chlorpyrifos, HLM is human liver microsome, HP is human plasma, CVs is coefficients of variation, HK_AF_ is the chemical-specific adjustment factor (CSAF) for human variability in toxicokinetics of chlorpyrifos, MPL is liver microsomal protein scaling factor, ISEF is the intersystem extrapolation factor for each CYP derived based on differences in activity between Supersomes^™^ and HLMs by incubating them with each relevant CYP-specific probe substrate, BMD is benchmark dose, “X” means the approach was terminated

PBK-based reverse dosimetry linked with Monte Carlo simulations was applied to predict the effect of inter-individual human kinetic variation in CPF metabolism for the inter-individual differences in RBC AChE inhibition following CPF exposure. Two approaches were used to define the metabolic variation in the PBK model, namely a Supersome^™^-based PBK model approach and an HLM-based PBK model approach. The whole procedure consisted of the following steps: (1) Establishment of an in vitro concentration–response curve for CPO in the AChE inhibition assay using rhAChE, (2) Collection of kinetic parameters (the maximum velocity (*V*_max_) and *K*_m_) for each metabolic pathway for the two approaches either from literature or from experiments, (3) Development of PBK models, and performance of Monte Carlo simulations by including kinetic data obtained in step 2, and CVs for metabolism-related kinetic input parameter (*V*_max_, *K*_m_ for HLM and abundance of each CYP isoform for Supersome^™^) and calculation of the HK_AF_, including also the variability of other influential parameters (e.g. body weight, absorption rate constants (*k*a), fractional absorption (fa), in vivo unbound fraction of CPO in plasma (fuCPO _in vivo(plasma)_) and blood to plasma ratio of CPO (BPCPO)), 4) PBK model-based reverse dosimetry to extrapolate the in vitro concentration–response curve to in vivo dose–response curves, (5) BMD analysis of the predicted in vivo dose–response curves and evaluation. In step 3, inter-individual variation was introduced by including the CVs of the CYP abundances from the Simcyp Simulator (V18 Release1 Certara, Sheffield, UK), the CVs on HLM-related kinetic data (*n* = 30) from Smith et al. (2011), and the CVs on plasma-related kinetic data (*n* = 25) from the present study. The CVs of other influential parameters was included as described in the section “2.5.4 Monte Carlo simulations”. The outline of the whole procedure is schematically presented in Fig. 2.

### In vitro AChE inhibition assay to derive CPO concentration–response curves

In the present study, the inhibitory effect of the active CPF metabolite CPO on RBC AChE was measured using rhAChE, as a proxy for RBC AChE, using the method previously published (Zhao et al. 2021), based on the method from Ellman et al. (1961). The working solutions were prepared by diluting a series of increasing concentrations of CPO (in ethanol) 50-fold in 100 mM sodium phosphate (pH 7.4) containing 0.1 mg/ml BSA. In a similar fashion, a 5000 µM CPO solution in ethanol (positive control) and a 100% ethanol solution (solvent control) was diluted. The rhAChE activity experiment was conducted in 96-well plates. In each well, 44 µl 100 mM sodium phosphate (pH 7.4) containing 0.1 mg/ml BSA, with 5 µl CPO working solution (final concentrations 0.05, 0.1, 0.5, 1, 2.5, 5, 10, 25 and 50 nM), or 5 µl positive control (CPO at a final concentration of 10,000 nM) or 5 µl solvent control (resulting in a final ethanol concentration of 0.2%) was added. The inhibition reaction was initiated by adding 1 µl rhAChE, resulting in a total incubation volume of 50 µl. The whole plate was incubated at 37 ℃ for 15 min. Subsequently, the inhibition reaction was terminated by adding 150 µl reaction reagents (mixture of ATC at a final concentration of 150 µM and DTNB at a final concentration of 75 µM) into each well to yield a final volume of 200 µl in each well (final enzyme concentration is 0.6 mU/ml). Finally, the solutions in the 96-well plate were measured continuously for 10 min at an absorbance of 412 nm at 37 ℃ to quantify the rhAChE activity.

The rhAChE activity was expressed as the remaining rhAChE activity relative to the solvent control (100% activity) and the positive control (0% activity) based on Eq. 1:

1 $${\text{rhAChE activity}}\% = \frac{{A412\left( {t10 - t0} \right){\text{Test compound}} - A412\left( {t10 - t0} \right){\text{Positive control}}}}{{A412\left( {t10 - t0} \right){\text{Solvent control}} - A412\left( {t10 - t0} \right){\text{Positive control}}}} \times 100\% ,$$

where the *A*412(*t*10–*t*0)Test compound is the change in the absorbance at *A*412 nm between 0 and 10 min for the test compound, the *A*412(*t*10–*t*0)Positive control is the change in the absorbance at *A*412 nm between 0 and 10 min for the CPO sample at the final concentration of 10,000 nM, and the *A*412(*t*10–*t*0)Solvent control is the change in the absorbance at *A*412 nm between 0 and 10 min for the 0.2% ethanol sample.

Concentration–response curves were constructed and the half maximal inhibitory concentrations (IC50) for CPO were derived, applying a non-linear regression model for Dose Response-Inhibition-Variable, with the equation log(inhibitor) vs. response-variable slope (four parameters) in GraphPad Prism 5, version 5.04 (GraphPad software, San Diego California USA), with 95% confidential interval.

### Collection of in vitro kinetic parameters for the PBK models

#### Supersome^™^-based PBK model

Metabolic kinetic constants (*V*_max_ and *K*_m_) of the hepatic CYP-catalyzed biotransformation of CPF (pathway 1 and pathway 2) (Fig. 1) were generated using the four CYP isoform Supersomes^™^ (CYP1A2, CYP2B6, CYP2C19 and CYP3A4) known to be the most dominant CYPs in the metabolism of CPF on the basis of the results from Foxenberg et al. (2007). CYP3A5 and CYP3A7 that were included in Foxenberg et al. (2007) were not included in the present study due to their limited contribution to the conversion of CPF. The in vitro kinetic incubations were conducted based on the method reported by Foxenberg et al. (2007) with some modifications. In detail, preliminary experiments were performed first to optimize the formation of metabolites to be linear with time and amount of Supersome^™^ protein (data not shown) for each CYP seperately.

The final incubations were carried out in 50 mM Tris–HCl (pH 7.4) containing 5 mM MgCl_2_, 1 mM EDTA (as an A-esterase PON1 inhibitor) (Bizoń and Milnerowicz 2018), 50 µM iso-OMPA (as a B-esterases inhibitor) (Lane et al. 2006), Supersome^™^ isoform (at final concentration of 0.005 nmol CYP/ml for 1A2, 0.001 nmol CYP/ml for 2B6, 0.01 nmol CYP/ml for 2C19, and 0.01 nmol CYP/ml for 3A4) and CPF at different final concentrations (0.05, 0.1, 0.25, 0.5, 1, 5 and 10 µM for 1A2, 0.05, 0.1, 0.25, 0.5, 1, 5 10 and 25 µM for 2B6, 1, 2.5, 5, 10, 25 and 50 µM for 2C19, and 0.5, 1, 2.5, 5, 10, 25 and 50 µM for 3A4), which were added from 100 times concentrated stock solutions in ACN. After 1 min preincubation in a 37 °C water bath, 5 µl NADPH (final concentration 1 mM) was added to the incubation system. Control incubations were performed by replacing NADPH with the same amount of Tris–HCl (pH 7.4). The total volume of the incubation mixtures was 200 µl. The incubations lasted for 2 min at 37 °C and were terminated by addition of 50 µl ice cold ACN and subsquently put on ice. After that, samples were centrifuged at 16,000 g (4 ℃) for 5 min and supernatants of samples were separated into two equal portions for Ultra-Performance Liquid Chromatography-Photodiode Array (UPLC-PDA) and Liquid Chromatography Mass Spectrometry (LC–MS/MS) analysis, respectively. For the samples that were measured by UPLC-PDA for quantification of TCPy formation, no further dilution was required, but for samples that were analyzed by LC–MS/MS to quantify CPO formation (limit of detection (LOD) = 3.1 nM), the ones that originally contained 2.5, 5, 10, 25, 50 µM of CPF were diluted 2.5×, 5×, 10×, 25× and 50×, respectively, in a mixture of ACN and 50 mM Tris–HCl (pH 7.4) (ratio 1:4, v/v).

To normalize for differences in intrinsic CYP activity between Supersome^™^ and pooled HLMs and enable scaling of the activities to the in vivo situation, CYP-specific ISEF values need to be established. The ISEF determination for each CYP was performed using Supersome^™^ and pooled HLM-derived *V*_max_ values for metabolite formation of probe substrates (specifically metabolized by a single CYP), being phenacetin (CYP1A2), bupropion (CYP2B6), (S)-mephenytoin (2C19) and testosterone (3A4) (Chen et al. 2011; Faucette et al. 2000). The incubation conditions used for these studies are summarized in Table 1. These incubation conditions were optimized and selected with respect to linearity for the formation of the metabolite of the CYP-specific probe substrate using both Supersome^™^ and pooled HLMs (data not shown). The incubation method was similar as used for the CPF kinetic incubation assay described above, except that certain incubation conditions were replaced by the conditions listed in Table 1. The samples obtained were analyzed by UPLC-PDA for acetaminophen, 4-hydroxymephenytoin, and 6β-hydroxytestosterone formation catalyzed by 1A2, 2C19 and 3A4, or LC–MS/MS for (±)-hydroxybupropion formation catalyzed by 2B6. For the samples that were analyzed by LC–MS/MS for quantification of (±)-hydroxybupropion formation, the ones that contained 100, 250, 500, 1000, 1500 and 2000 µM bupropion were further diluted 2×, 5×, 10×, 20×, 40× and 40×, respectively, in a mixture of ACN and 50 mM Tris–HCl (pH 7.4) (ratio 1:4, v/v). LC–MS/MS was used for quantification of (±)-hydroxybupropion formation, because it is allowing low levels of (±)-hydroxybupropion (LOD = 15.5 nM) to be detected.

**Table 1:** Selected probe substrate for each CYP and their corresponding incubation conditions used to derive ISEF

Pooled HLM					
CYP isoform	Probe substrate	Specific metabolite	Pooled HLM concentration (mg/ml)	Substrate concentration range (µM)	Incubation time (min)
1A2	Phenacetin	Acetaminophen	0.5	10, 25, 50, 100, 250, 500	40
2B6	Bupropion	(±)-Hydroxybupropion	0.05	5, 10, 25, 50, 100, 250, 500, 1000, 1500, 2000	20
2C19	(S)-mephenytoin	4-Hydroxymephenytoin	0.5	5, 10, 25, 50, 100, 300, 500	40
3A4	Testosterone	6β-Hydroxytestosterone	0.2	10, 25, 50, 100, 250, 500	5

Supersome^™^					
CYP isoform	Probe substrate	Specific metabolite	Supersome^™^ concentration (pmol CYP/ml)	Substrate concentration range (µM)	Incubation time (min)
1A2	Phenacetin	Acetaminophen	0.01	10, 25, 50, 100, 250, 500	20
2B6	Bupropion	(±)-Hydroxybupropion	0.01	5, 25, 50, 100, 250, 500, 1000, 2000	10
2C19	(S)-mephenytoin	4-Hydroxymephenytoin	0.01	5, 10, 25, 50, 100, 300, 500	20
3A4	Testosterone	6β-Hydroxytestosterone	0.005	10, 25, 50, 100, 250, 500	2

For the hepatic PON1-mediated detoxification of CPO (pathway 3), the previously established kinetic parameters (*V*_max_ and *K*_m_) by Zhao et al. (2019) using pooled HLMs were used.

The metabolic parameters of the PON1-catalyzed detoxification of CPO in plasma (pathway 4) were obtained by incubating different concentrations of CPO with 25 individual HP samples. Pilot studies were conducted to optimize the metabolite formation to be linear with the in vitro plasma concentration and time of incubation (data not shown). Briefly, the incubations were carried out in 50 mM Tris–HCl (pH 7.4) containing 2 mM CaCl_2_ and CPO at final concentrations of 10, 50, 100, 250, 500 and 1000 µM (added from 100 times concentrated stock solutions in ACN). After 1 min preincubation in a shaking 37 °C water bath, 1 µl of individual HP was added into the incubation system. Control incubations were performed by replacing plasma with the same amount of 50 mM Tris–HCl (pH 7.4). The total volume of the incubation mixtures was 200 µl. The incubation lasted for 1.5 min at 37 °C and was terminated by addition of 200 µl ice cold ACN and subsquently put on ice. Samples were subsequently centrifuged at 16,000 g (4 ℃) for 5 min and analyzed by UPLC-PDA.

#### HLM-based PBK model

In total, pathway 1 of the HLM-based PBK model contains two scenarios. For scenario 1 (non-biphasic), the previously published kinetic parameters (Sams et al. 2004) for bioactivation of CPF obtained using incubations with pooled HLMs were employed in the present study (Fig. 2). In this study (Sams et al. 2004), the CPF concentration range used (3–100 µM) covered the Km of CYP3A4, as reflected by consistency in the apparent Km obtained in pooled HLMs from Sams et al. (2004) (29.8 µM) and that in Supersome^™^ CYP3A4 reported by Foxenberg et al. (2007) (27.3 µM) and obtained in the present study (29.8 µM) (Table 4). On the other hand, this CPF concentration range far exceeds the Km of CYP1A2, CYP2B6, CYP2C19 reported to amount to 0.38 µM, 0.81 µM, 1.23 µM (Foxenberg et al. 2007), or 0.61 µM, 0.14 µM, 1.89 µM (the present study, Table 4), respectively. Use of CPF concentrations far exceeding the Km may inadequately describe the high affinity kinetic phase mediated by these high affinity (low Km) CYPs at low concentrations (< 3 µM). Therefore, to identify the importance of including such high affinity kinetics in PBK model descriptions of the bioactivation of CPF to CPO, a second kinetic scenario (biphasic) was defined for the HLM approach (Fig. 2). This biphasic scenario includes a high affinity (low *K*_m_) kinetic phase in addition to the low affinity (high *K*_m_) kinetic phase. To describe the high affinity kinetics of the HLM, the apparent *K*_m_ and *V*_max_ defined at the low concentration range of 0.02 µM to 10 µM in pooled HLMs as reported by Buratti et al. (2003) were used. In this way, the biphasic kinetics of the bioactivation of CPF at both low and high concentration ranges could be fully captured. Application of biphasic kinetics is further supported by the scaled Supersome^™^ kinetic data presented below in the “Result” section. Also, Ma and Chambers (1995) reported biphasic kinetics for bioactivation of CPF by rat liver microsomes, reflected by a high affinity and a low affinity metabolic phase, with the Km value for the high affinity phase being 50-fold lower than that for the low affinity phase.

To describe the detoxification of CPF by pooled HLMs (Pathways 2 in Fig. 1), the previously published monophasic kinetic parameters of Sams et al. (2004) were used. Applying monophasic kinetics, and not biphasic kinetics as applied for Pathway 1, is based on the data from the present study (see “Result” section), showing that for this reaction the conversion at low concentrations was not dominated by a high affinity CYP.

In the HLM-based PBK model, for the PON1-mediated detoxification of CPO in liver and plasma (pathway 3 and pathway 4), respectively, the same kinetic parameters of pathway 3 and pathway 4 as described in the section “Supersome^™^-based PBK model” were used.

#### Determination of total protein concentration in individual human plasma samples

The total protein concentration of the 25 individual HP samples was measured using a Pierce^™^ BCA protein assay kit (ThermoFisher Scientific 2020), enabling scaling of the kinetic parameters measured for pathway 4 from per mg protein to per ml plasma. The experiment was performed based on the manufacturer’s protocol. In detail, 25 µl sample or protein standard solution at concentrations of 0.025, 0.125, 0.25, 0.5, 0.75, 1 and 2 mg/ml were added and incubated with 200 µl working reagents at 37 ℃ for 30 min in a 96-well plate. After that, the plate was cooled to room temperature, and each sample and each protein standard was measured at 562 nm (absorbance). The total protein concentration of each unknown sample was calculated based on the calibration curve (protein concentration versus 562 nm absorbance value) generated with the protein standards.

#### Calculation of kinetic parameters

As mentioned before, the in vitro apparent kinetic parameters (*V*_max_ and *K*_m_) for pathway 1 and pathway 2 in the HLM-based approach, and pathway 3 in both approaches were all obtained directly from reported studies using pooled HLMs (Buratti et al. 2003; Sams et al. 2004; Zhao et al. 2019). Therefore, no extra calculations were required to define these parameters. The kinetic parameters for the ISEF calculation, pathway 1 and 2 in the Supersome^™^-based approach, and pathway 4 in both approaches (25 individuals) were all obtained by fitting the experimental data to the Michaelis–Menten model using GraphPad (GraphPad Prism 5.0 software, San Diego, CA, USA). It should be noted that the kinetic parameters for pathway 4 used in the two approaches were calculated as mean of the kinetic parameters (*V*_max_ and *K*_m_) obtained using 25 individual HP samples (with correction for total protein concentration of each plasma sample).

#### Quantification of metabolites of CPF, CPO and probe substrates by UPLC-PDA and LC–MS/MS

The analysis method, gradient elution and retention time for each compound by UPLC-PDA or LC–MS/MS are described in Supplementary material I.

### Development of the PBK model, Monte Carlo simulations and establishment of chemical-specific adjustment factors (CSAFs)

#### Model development

In the present study, our previously developed PBK model for CPF (predicting total blood concentrations of CPO) in Caucasian (Zhao et al. 2019) was used as a starting point for the PBK model, which is used for evaluation of inter-individual variation in metabolism of CPF and the consequences of this variation for the induced RBC AChE inhibition. Compared to the PBK model of Zhao et al. (2019), only the fractional absorption was changed to 0.462, which is the mean value of reported fa values from Timchalk et al. (2002) (fa = 0.224) and Nolan et al. (1984) (fa = 0.7), while for the other model parameters (non-kinetic), the mean values as reported in Zhao et al. (2019) were used.

Compared to the model of Zhao et al. (2019), in the Supersome^™^-based PBK model approach, the kinetic parameters of pathway 1 and pathway 2 were replaced from HLM-derived values to Supersome^™^-derived values, while the kinetic parameters of pathway 3 were kept the same. For pathway 4, instead of using the reported kinetic parameters from Mosquin et al. (2009), the calculated mean values of kinetic parameters obtained based on 25 individual HP samples derived by the present study were used. The kinetic equation that was applied for each metabolic pathway in the PBK model is indicated below:

For pathway 1 and pathway 2, the overall kinetic parameters of four CYP isoforms (CYP1A2, CYP2B6, CYP2C19 and CYP3A4) in liver were described by Eq. 2 (Foxenberg et al. 2007):

2 $$v_{{{\text{overall supersome}}^{{{\text{TM}}}} }} = \sum \left( { \frac{{V\max_{i} \times \left[ S \right]}}{{\left( {Km_{i} + \left[ S \right]} \right)}} } \right),$$

where *v*_overall supersome_^™^ is the overall (summed) in vivo metabolic rate for all four CYP isoforms (umol/h); i represents different CYP isoforms (CYP1A2, CYP2B6, CYP2C19 and CYP3A4), [S] is the blood concentration of CPF (µM), *V*_maxi_ and *K*_mi_ are in vivo maximum velocity (umol/h) and the Michaelis–Menten constant (µM) for each respective CYP isoform in either pathway 1 or pathway 2. The in vivo *K*_mi_ values were assumed to be equal to the apparent *K*_mi(app)_ values (µM) obtained in the in vitro incubations, while the in vivo *V*_maxi_ for each CYP isoform was derived from their corresponding apparent CYP isoform *V*_maxi(app)_ (pmol/min/pmolCYP) using Eq. 3:

3 $$V\mathop {\max }\nolimits_{{_{i} }} = \frac{{V\max_{{i\left( {app } \right)}} \times CYP_{i} {\text{abundance}} \times ISEF \times 60 \times MPL \times 1000 \times VL}}{1,000,000},$$

In the equation CYP_i_ abundance is the average endogenous abundance (pmolCYP/mg microsomal protein, representing the amount of the respective CYP per mg of microsomal protein) of CYP isoform i (CYP1A2 = 52, CYP2B6 = 16, CYP2C19 = 5.4 and CYP3A4 = 137) in HLMs obtained from Simcyp (Simcyp Simulator V18 Release 1, Certara, Sheffield, UK), 60 is to account for the unit change from min to h, MPL is the microsomal protein yield of 32 mg/g liver (Barter et al. 2007), 1000 is to account for the unit change from g to kg, VL is the weight of the liver tissue (kg), 1,000,000 is to account for the unit change from pmol to µmol, and ISEF_i_ is the intersystem extrapolation factor for the CYP isoform i, which were determined based on the ratio between the apparent Vmax of metabolite formation for each CYP-specific probe substrate in pooled HLMs and the respective Supersome^™^ (with correction for the average CYP abundance in HLM), according to Eq. 4:

4 $$ISEF_{i} = \frac{{V\max {\text{probe}} _{{z\left( {pooled HLMs} \right)}} \times 1000 }}{{V\max probe _{{z\left( {{\text{Supersomes}}^{{{\text{TM}}}} } \right)}} \times CYP_{i } \,{\text{abundance}}}},$$

In which z represents the probe substrate (phenacetin, bupropion, (S)-mephenytoin and testosterone), *V*_max_ probe _z (pooled HLMs)_ is the apparent maximum rate (nmol/min/mg microsomal protein) for the kinetic conversion of probe substrate z obtained using pooled HLMs, and Vmax probe _z (Supersomes_™_)_ is the apparent maximum rate (pmol/min/pmol CYP) for the kinetic conversion of probe substrate z obtained using Supersomes^™^, 1000 is to account for the unit change from nmol/min/mg microsomal protein to pmol/min/mg microsomal protein, and the CYP_i_ abundance (pmol CYP/mg microsomal protein) is defined as above.

For pathway 3 and pathway 4, the metabolic reactions were described by Eq. 5:

5 $$\mathop v\nolimits_{HLM} = \frac{{V\max_{ HLM} \times \left[ S \right] }}{{\left( {Km_{ HLM} + \left[ S \right]} \right) }} {\text{or}}\,\,v_{HP} = \frac{{V\max_{HP} \times \left[ S \right] }}{{\left( {Km_{HP} + \left[ S \right]} \right) }} ,$$

where *v*_HLM_ and *v*_HP_ represent the in vivo rate of the metabolic reaction (µmol/h) of pathway 3 or pathway 4, S represents the blood concentration of CPO (µM), *V*max_HLM_ and *K*m_HLM_ are the in vivo maximum velocity (µmol/h) and the Michaelis–Menten constant (µM) of pathway 3, and *V*max_HP_ and *K*m_HP_ are the in vivo maximum velocity (µmol/h) and the Michaelis–Menten constant (µM) of pathway 4.

In pathway 3, the in vivo *V*max_HLM_ (µmol/h) was extrapolated from its corresponding apparent *V*max_HLM(app)_ (in nmol/min/mg microsomal protein) using Eq. 6:

6 $$V\mathop {\max }\nolimits_{HLM} = \frac{{V\max _{{HLM({\text{app}})}} \times 60 \times MPL \times 1000 \times VL}}{1000},$$

In this equation, 60 is to account for the unit change from min to h, MPL is the microsomal protein yield of 32 mg/g liver (Barter et al. 2007), 1000 is to account for the unit change from g to kg or nmol to µmol, and VL is the weight of the liver tissue (kg).

In pathway 4, the in vivo *V*max_HP_ (µmol/h) was extrapolated from its corresponding mean of the apparent *V*max_HP(app)_ (nmol/min/ml plasma) of 25 individuals HP using Eq. 7:

7 $$V\mathop {\max }\nolimits_{HP} = \frac{{V\max_{{HP({\text{app}})}} \times 60 \times 1000 \times VB \times 0.55}}{1000},$$

In this Equation, 60 is to account for the unit change from min to h, 1000 is to account for the unit change from mL to L, VB is the volume of the blood (L), 0.55 represents the plasma to blood volume ratio used to scale the unit of *V*max of PON1-mediated conversion (Pathway 4) from per ml plasma to the whole plasma of the blood compartment, based on the fact that the PON1 is present in plasma and the human plasma volume amounts to 55% of the blood volume (Li et al. 2005; O’Neil 1999). 1000 is to account for the unit change from nmol to µmol.

The in vivo Km values for both pathway 3 and pathway 4 were assumed to be equal to their corresponding apparent Km values obtained in the in vitro incubations.

For the development of the HLM-based PBK model, scenario 1 (non-biphasic) was excluded due to the inadequate kinetic description of CPO formation at low dose levels (see “Results” section “Bioactivation and detoxification of CPF in Supersome^TM^ enzymes”). For scenario 2 (biphasic), the kinetic parameters of pathway 1 used in the original model described by pooled HLMs (Zhao et al. 2019) were replaced by reported kinetic parameters for the high affinity phase (Buratti et al. 2003) and the low affinity phase (Sams et al. 2004), while those for pathway 2 were replaced by data from Sams et al. (2004). The kinetic parameters of pathway 3 and pathway 4 used for the HLM-based PBK model were the same as the ones used for the Supersome^™^-based PBK model. The kinetic equation for each pathway in the human model was described as presented below:

The total velocity for hepatic bioactivation of CPF (pathway 1) was described by biphasic kinetics using Eq. 8 (Venkatakrishnan et al. 2001):

8 $$v_{{\text{overall HLM}}} = \frac{{V\max_{{\text{high affnity}}} \times \left[ S \right] }}{{\left( {Km_{{\text{high affinity}}} + \left[ S \right]} \right) }} + \frac{{V\max_{{\text{low affinity}}} \times \left[ S \right] }}{{\left( {Km_{{\text{low affnity}}} + \left[ S \right]} \right) }} ,$$

where *v*_overall HLM_ (µmol/h) is the overall in vivo metabolic rate of pathway 1 (including both the high and low affinity phase); [S] is the blood concentration of CPF (µM), *V*max_high affinity_ and *K*m_high affinity_ are the in vivo maximum velocity (µmol/h) and the Michaelis–Menten constant (µM) of the high affinity phase, while *V*max_low affinity_ and *K*m_low affinity_ are those of the low affinity phase. The in vivo *K*m values were assumed to be equal to the apparent *K*m values obtained from the in vitro experiments, and the in vivo *V*max for both the high and low affinity phase were extrapolated from their corresponding apparent *V*max values obtained in the in vitro experiments using Eq. 6.

The hepatic reaction of pathway 2 was described using Eq. 5 and Eq. 6.

The metabolic reactions of pathway 3 and pathway 4 were described in the same way as done for the Supersome^™^-based PBK model approach (see section “Model development”).

#### Model evaluation

The performance of the Supersome^™^-based PBK model and the HLM-based PBK model (biphasic) were evaluated by comparing their predicted time-dependent TCPy and CPF blood concentrations including the maximum total (bound and unbound) blood concentration (*C*_max_) or time-dependent blood concentration, and the cumulative time-dependent urinary TCPy excretion with corresponding available in vivo data (Drevenkar et al. 1993; Nolan et al. 1984; Timchalk et al. 2002) upon similar dosing regimens or estimated dose level. The time-dependent CPF concentration data from Drevenkar et al. (1993) relate to concentrations in plasma. To allow comparison to PBK model-based predicted blood concentrations, these plasma concentrations of CPF were converted to a blood concentration by multiplying with the blood to plasma ratio for CPF of 1.3 (the ratio of the CPF concentration in blood to the CPF concentration in plasma), which was estimated using Simcyp (2016a) based on CPF LogP of 4.784 (ChemAxon) and a fraction unbound of 0.021 for plasma (Simcyp 2016b)). Drevenkar et al. (1993) reported these time-dependent CPF concentration for a poisoning victim who drunk 30–60 ml of pesticide product Chromorel D^®^. Given that the concentration of CPF in Chromorel D^®^ is 500 g/l (see https://www.agroklub.ba/poljoprivredni-oglasnik/oglas/chromorel-d/16769/), the CPF intake for this victim was assumed to be 30–60 ml of 500 g CPF/l corresponding to 15,000 to 30,000 mg CPF. The corresponding estimated dose was calculated on the basis of a body weight of 70 kg, to amount to 214–429 mg/kg bw.

#### Sensitivity analysis

The sensitivity analysis was performed as described by Zhao et al. (2019) to identify model parameters that influence the predicted free blood *C*_max_ of CPO at single oral dose levels of CPF (0.5 mg/kg bw and 180 mg/kg bw).

#### Monte Carlo simulations

To include the inter-individual variation in the biotransformation reactions of CPF in human in the model-based predictions, a Monte Carlo simulation using the CV of the kinetic parameters for each metabolic pathway in the Supersome^™^-based PBK model and the HLM-based PBK model (biphasic) was incorporated into the PBK models. More specifically, in the Supersome^™^-based PBK model, kinetic variation in pathway 1 and 2 was described based on kinetic data (*V*max and *K*m) from Supersomes^™^ together with reported CVs in CYP abundance, while in the HLM-based PBK model (biphasic), the variation in these two pathways was described based on pooled HLM kinetic data (*V*max and *K*m) together with the reported CV values for these pathways obtained from HLM kinetic data from 30 individuals (Smith et al. 2011). Both in the Supersome^™^-based and the HLM-based PBK model, variation in pathway 3 was based on pooled HLM kinetic data (*V*max and *K*m) together with the reported CV for this pathway observed in 30 individuals (Smith et al. 2011), while variation in pathway 4 was based on the mean values of kinetic data of CPO detoxification in plasma measured for 25 individual HP samples together with their corresponding CV values derived in the present study.

The input parameters (CYP abundance, *V*max and *K*m) were assumed to follow a lognormal distribution. Given that Berkeley Madonna only offers the ‘NORMAL’ distribution function for sampling random numbers and not a lognormal function, the mean (*µ*_x_) and standard deviations (*σ*_x_) from these lognormally distributed parameters (*V*max or *K*m or abundance of relative CYP) were first transformed to parameters following a normal distribution using Eq. 9 (Zhang et al. 2007):

9 $$\mu_{w} = \ln \left( {\frac{{\mu_{x} }}{{\sqrt {1 + CV_{x}^{2} } }}} \right)\, {\text{and}}\,\, \sigma_{w}^{2} = \ln \left( {1 + CV_{x}^{2} } \right) ,$$

where *µ*_x_ is the mean of *V*max or *K*m or abundance of the respective CYP, and CV_x_ is the coefficient of variation for each of the values, after which the exponential function (Eq. 10) on the normally distributed *µ*_w_ and *σ*_w_ was applied to obtain the lognormally distributed input parameters in the model (Li et al. 2017):

10 $$\log {\text{normal}} \left( {\mu_{x} ,\sigma_{x} } \right) = \exp \left( {{\text{Normal}} \left( {\mu_{w} ,\sigma_{w} } \right)} \right) .$$

A summary of the mean and the CV values that define the distributions for the input parameters *V*max, *K*m and CYP abundance used for the Monte Carlo simulation in the two models is shown in Table 2. It should be noted that the parameters obtained using pooled HLMs were considered to represent a mean value (Table 2). Different than CYP1A2 and CYP3A4 for which only one phenotype is known, more than one phenotype is known for CYP2B6 and CYP2C19. Therefore, in the Supersome^™^-based PBK model, the variation in the activity of CYP2B6 and CYP2C19 was characterized by considering different phenotype abundances and relative frequencies in the general population obtained from Simcyp (Simcyp Simulator V18 Release 1, Certara, Sheffield, UK) in the Monte Carlo analysis.

**Table 2:** A summary of the mean value and coefficient of variation (CV) for *V*max, *K*m, CYP abundance of each CYP phenotype and their relative frequency in the general population

**Supersome**^**™**^**-based PBK model**				
Pathway 1 and pathway 2 (CYP abundance)				
Parameter	Mean^a^	CV	Frequency	Reference
1A2 (EM^b^)	52.0	0.67	1.000	Simcyp^l^
2B6 (EM^b^)	17.0	1.22	0.890	Simcyp^l^
2B6 (PM^c^)	6.0	2.00	0.110	Simcyp^l^
2C19 (EM^b^)	4.4	0.71	0.590	Simcyp^l^
2C19 (PM^c^)	0.0	0.00	0.092	Simcyp^l^
2C19 (UM^d^)	8.7	0.71	0.318	Simcyp^l^
3A4 (EM^b^)	137	0.41	1.000	Simcyp^l^

Pathway 3 (*V*max, *K*m)				
Parameter	Mean	Reference	CV^k^	Reference
*V*max	37.980 ^e^	Zhao et al. (2019)	0.57	Smith et al. (2011)
*K*m	627.900 ^f^	Zhao et al. (2019)	0.39	Smith et al. (2011)

Pathway 4 (*V*max, *K*m)				
Parameter	Mean	Reference	CV	Reference
*V*max	1844.000^i^	Calculated^j^	0.29	Calculated^j^
*K*m	290.000^f^	Calculated^j^	0.33	Calculated^j^

**HLM-based PBK model (biphasic)**				
Pathway 1 (*V*max, *K*m)				
Parameter	Mean^k^	Reference	CV^k^	Reference
*V*max (high affinity)^g^	0.275	Buratti et al. (2003)	0.59	Smith et al. (2011)
Km (high affinity)^h^	0.270	Buratti et al. (2003)	0.61	Smith et al. (2011)
Vmax (low affinity)^e^	0.353	Sams et al. (2004)	0.59	Smith et al. (2011)
Km (low affinity)^f^	29.800	Sams et al. (2004)	0.61	Smith et al. (2011)

Pathway 2 (*V*max, *K*m)				
Parameter	Mean^k^	Reference	CV^k^	Reference
*V*max^e^	0.653	Sams et al. (2004)	0.53	Smith et al. (2011)
*K*m^f^	12.000	Sams et al. (2004)	0.89	Smith et al. (2011)

Pathway 3 & Pathway 4 (*V*max, *K*m)				
Same as the values reported in Supersome^TM^-based PBK model for Pathway 3, Pathway 4				

^a^pmol CYP/mg microsomal protein

^b^*EM* extensive metabolizer

^c^*PM* poor metabolizer

^d^*UM* ultra-rapid metabolizer

^e^*V*_max_ obtained using a high concentration range (3–100 µM) of CPF, in nmol/min/mg microsomal protein

^f^*K*_m_ obtained using a high concentration range (3–100 µM) of CPF, in µM

^g^*V*_max_ obtained using low concentration range (0.02–10 µM) of CPF, in nmol/min/mg microsomal protein

^h^*K*_m_ obtained using low concentration range (0.02–10 µM) of CPF, in µM

^i^nmol/min/ml plasma

^j^The value is calculated using the kinetic data from 25 individual HP samples characterized in the present study, the detailed results are presented in the section “Supplementary IV”

^k^Data obtained from Smith et al. (2011) for CV calculation, based on the fact that the metabolic reactions of CPF bioactivation, CPF and CPO detoxification are not age-dependent in HLM when expressed on the basis of per mg microsomal protein

^l^Simcyp (Simcyp Simulator V18 Release 1, Certara, Sheffield, UK)

In total 10,000 simulations were performed for the Monte Carlo analysis. In each simulation, the parameter values were randomly taken from the distributions of the input parameters. To avoid sampling unrealistic values, a minimum and maximum value for each parameter distribution was established by applying a cut-off value corresponding to ± 3 *σ*_w_ of the mean *µ*_w_ (Strikwold et al. 2017). It is of importance to mention that no correlation was assumed between different metabolic pathways of CPF in the present study. As mentioned above, some CYP isoforms contained different sub-phenotypes, therefore the Monte Carlo analysis for the Supersome^™^-based PBK model took different phenotypes and their corresponding frequencies (included in Table 2) in the general population into account. In total, there were six possible combinations of different CYP phenotypes (1A2EM, 2B6EM, 2C19EM, 3A4EM; 1A2EM, 2B6EM, 2C19PM, 3A4EM; 1A2EM, 2B6EM, 2C19UM, 3A4EM; 1A2EM, 2B6PM, 2C19EM, 3A4EM; 1A2EM, 2B6PM, 2C19PM, 3A4EM; 1A2EM, 2B6PM, 2C19UM, 3A4EM). To obtain the overall frequency distributions for the entire population, the six possible combinations were run independently for 10,000 runs, and their resulting distributions were corrected with the corresponding frequency of the phenotype of the respective CYPs in the general population (Table 2), and summed together at the end.

The HK_AF_ of CPF at a dose of 0.47 mg/kg bw was calculated as the ratio between the 95^th^ or 99^th^ percentile of CPO formation and the geometric mean (GM) for the whole population (IPCS 2005). Use of a dose level of 0.47 mg/kg bw for this calculation was based on the fact that it represents the benchmark dose lower confidence limit for 10% inhibition (BMDL_10_), which is used as the POD for setting a health based guidance value (USEPA 2014).

The sensitivity analysis revealed that in addition to the metabolism-related kinetic parameters, also other parameters (such as body weight, fraction of liver tissue (VLc), fraction of blood (VBc), cardiac output (QC), fraction of blood flow to liver (QLc), fraction of blood flow to richly perfused tissue (QRc), ka, MPL, fa, fuCPO _in vivo(plasma)_, and BPCPO) have a substantial impact on the C_max_ of CPO and hence can contribute to the overall inter-individual variability in kinetics, thereby potentially influencing HK_AF_ values. Therefore, the variability in these additional influential parameters was also included in the Monte Carlo simulations. These simulations were conducted in a similar way as described above. It is good to mention that the variability of parameters VLc, VBc, QC, QLc, QRc are described by the variability of the parameter body weight in the Monte Carlo simulations because they are covariant (VLc, VBc, QC, QLc, QRc are calculated based on body weight), thus no CV and mean values of these parameters were included in Table 3. The mean value for each of the other influential parameters (Table 3) was directly obtained or calculated based on literature reported values (Barter et al. 2007; Bouchard et al. 2005; Brown et al. 1997; Heilmair et al. 2008; Nolan et al. 1984; Timchalk et al. 2002), or predicted by Simcyp (Simcyp Simulator V18 Release 1, Certara, Sheffield, UK). Their corresponding CVs were either obtained or calculated based on literature reported data (Barter et al. 2007; Nolan et al. 1984; Timchalk et al. 2002). When a CV was not available or could not be calculated, a default value of 0.3 was used to represent a moderate level of variation (Covington et al. 2007). Given that BPCPO was predicted based on fuCPO_in vivo(plasma)_ in Simcyp (Simcyp Simulator V18 Release 1, Certara, Sheffield, UK), the variation in BPCPO was assumed to be correlated with the variation in fuCPO _in vivo(plasma)_.

**Table 3:** A summary of the mean values and coefficients of variation (CV) for influential PBK model parameters other than kinetic parameters for metabolism

Supersome^™^—based and HLM-based PBK model				
Parameter	Mean	Reference	CV	Reference
BW^a^	70	Brown et al. (1997)	0.3	Default^f^
ka^b^	0.46	Bouchard et al. (2005)	0.3	Default^f^
fa^c^	0.46	Nolan et al. (1984; Timchalk et al. (2002)	0.73 g	Nolan et al. (1984, Timchalk et al. (2002)
MPL^d^	32	Barter et al. (2007)	0.46	Barter et al. (2007)
fuCPO_in vivo(plasma)_	0.15	Heilmair et al. (2008)	0.3	Default^f^
BPCPO	2.7	Simcyp^e^	0.3	Default^f^

^a^kg

^b^/h

^c^Mean value of reported absorption fraction fa values from Timchalk et al. (2002) (fa = 0.224) and Nolan et al. (1984) (fa = 0.7)

^d^mg microsomal protein/g liver

^e^Simcyp (Simcyp Simulator V18 Release 1, Certara, Sheffield, UK)

^f^Due to absence of a CV, a default value of 0.3 was used to represent a moderate level of variation (Covington et al. 2007)

^g^CV was calculated based on reported fa values from Timchalk et al. (2002) (fa = 0.224) and Nolan et al. (1984) (fa = 0.7). Given that the application of 3 σw of the mean fa value resulted in an unrealistic fa value higher than 1, the maximum upper cut-off value was set equal to1

#### Reverse dosimetry to extrapolate in vitro AChE inhibition data to in vivo dose–response curves

In the present study, PBK model-based reverse dosimetry was applied to translate the in vitro rhAChE inhibition concentration–response curve into in vivo RBC AChE inhibition dose–response curves first for the average adult population. To this end, in vitro effective concentrations of CPO were set equal to the in vivo free blood C_max_ values of CPO in the PBK model according to the following Eq. 11 (Shi et al. 2020):

11 $$C _{{\text{Total in vitro}}} \times {\text{fuCPO}}_{{\text{in vitro}}} = C _{{\text{Total in vivo}}} \times {\text{fuCPO}}_{{{\text{in vivo}}\left( {{\text{blood}}} \right)}} ,$$

Herein the *C*_total in vitro_ represents the total concentration of CPO in the in vitro assay and fuCPO_in vitro_ is the unbound fraction of CPO in the vitro assay. In the present study, the fuCPO_in vitro_ was assumed to be 1, which is based on the observation from Heilmair et al. (2008) that the presence of only a low level (0.1 mg/ml) of albumin in the in vitro medium as routinely used to stabilize the enzyme does not substantially affect the free fraction of CPO. *C*_total in vivo_ represents the total concentration of CPO in human blood. To obtain the corresponding unbound blood concentrations, the *C*_total in vivo_ was multiplied with the unbound fraction of CPO in blood (fuCPO_(in vivo(blood)_), which was obtained by dividing the unbound fraction of CPO in plasma (fuCPO_(in vivo(plasma)_) by the ratio of the CPO concentration in blood to the CPO concentration in plasma, the CPO blood to plasma ratio (BPCPO), as below (Eq. 12) (Shi et al. 2020):

12 $${\text{fuCPO}}_{{{\text{in vivo}}\left( {{\text{blood}}} \right)}} = \frac{{{\text{fuCPO}}_{{{\text{in vivo}}\left( {{\text{plasma}}} \right)}} }}{{{\text{BPCPO}}}} ,$$

The BPCPO value of 2.7 was estimated by Simcyp (Simcyp 2016a) based on the logP value (3.89) of CPO from ChemAxon (ChemAxon) and the unbound fraction of CPO in plasma (fuCPO_in vivo(plasma)_ = 0.15) from Heilmair et al. (2008).

The external dose values thus obtained together with corresponding inhibitory effects were used to construct RBC AChE inhibition dose–response curves for the average population. The dose–response curves for the most sensitive individuals (99th percentile) and least sensitive individuals (1st percentile) were derived by applying the obtained HK_AF_ (99th percentile) or the ratio between the 1st percentile and the GM of CPO formation distribution of the whole population to the dose–response curve of the average population.

#### BMD analysis and evaluation of the PBK model-based reverse dosimetry predictions

A BMD analysis was performed to derive a POD from the predicted in vivo dose–response curves obtained for the average population. In the present study, the BMDL_10_ values were used as POD based on the fact that the United States Environmental Protection Agency (USEPA) also used a 10% effect level to define a health based guidance value (USEPA 2011, 2014). BMD analysis was carried out using the Benchmark Dose Software version 3.1.2 (USEPA 2019), with exponential or hill models because of their adequacy in predicting continuous data. The BMDL_10_ value with the lowest AIC was chosen and further evaluated by comparing the obtained values with reported BMDL_10_ by USEPA (2011, 2014).

## Results

### In vitro AChE inhibition concentration–response curve

**Fig. 3:**
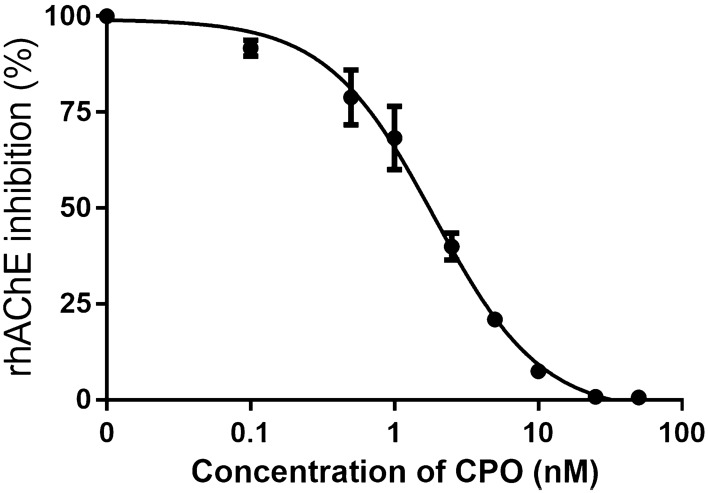
Effect of increasing concentration of CPO on recombinant human acetylcholinesterase (rhAChE) activity at 37 ℃. Each value represents the mean ± SD of five independent experiments

The CPO concentration-dependent inhibition of rhAChE activity is shown in Fig. 3. The 50% inhibition of enzyme activity occurred at a CPO concentration of 1.89 nM, with the 95% confidence interval ranging from 1.65 nM to 2.16 nM.

### Kinetic data, ISEF and total protein concentration

#### Bioactivation and detoxification of CPF by Supersome^™^ enzymes

The CPF concentration-dependent increase in the rate of bioactivation of CPF to CPO and detoxification of CPF to TCPy obtained with different Supersomes^™^ is presented in Supplementary material II. The apparent *V*max, *K*m and the CE (calculated as *V*max/*K*m) derived from these in vitro data are shown in Table 4. Overall, the Supersome^™^ data reveal that the four CYPs known to be active in CPF to CPO conversion vary in their velocity and affinity, with CYP2B6 showing the highest CE for bioactivation of CPF, and CYP2C19 showing the highest CE in terms of detoxification. CYP3A4 has the lowest CE for both pathways.

**Table 4:** Kinetic parameters for biotransformation of CPF in incubations with Supersomes^™^, and ISEFs for hepatic CYP-mediated biotransformation of probe substrates as determined based on incubations with pooled HLMs or Supersomes^™^

Biotransformation of CPF				
	CYP1A2	CYP2B6	CYP2C19	CYP3A4
Pathway 1				
*K*m(app)^a^	0.61	0.14	1.89	29.77
*V*max(app)^b^	3.96	7.76	2.74	17.78
CE^c^	6.49	55.43	1.45	0.60
Scaled *V*max(app)^d^	51.81	203.94	10.81	910.77
Scaled CE^e^	84.93	1456.70	5.71	30.59

Pathway 2				
*K*m(app) ^a^	1.25	1.28	1.37	18.13
*V*max(app) ^b^	2.96	5.49	17.51	23.86
CE^c^	2.37	4.29	12.78	1.32
Scaled *V*max(app)^d^	38.73	144.28	69.06	1222.21
Scaled CE^e^	30.98	112.72	50.41	67.41

ISEF determination				
	CYP1A2	CYP2B6	CYP2C19	CYP3A4
Probe substrate	Phenacetin	Bupropion	(S)-mephenytoin	Testosterone
CYP abundance^f^	52.00	15.80	5.40	137.00
*V*max _(pooled HLMs)_^g^	0.15	0.31	0.02	17.29
*V*max _(Supersome_^™^_)_^h^	39.06	40.77	20.59	1179
ISEF^i^	0.07	0.48	0.21	0.11

Data represent mean of two experiments for each parameter

^a^µM

^b^pmol/min/pmolCYP

^c^*CE* in vitro catalytic efficiency (µl/min/ pmol CYP) calculated as *V*max _(app)_/Km _(app)_

^d^Scaled *V*max (umol/h), calculated based on Eq. 3

^e^Scaled catalytic efficiency (l/h), calculated as scaled *V*max _(app_)/*K*m _(app)_

^f^CYP abundance (pmol CYP/mg microsomal protein) is the average endogenous abundance of each CYP isoform in HLM, which is obtained from Simcyp (Simcyp Simulator V18 Release 1, Certara, Sheffield, UK)

^g^nmol/min/mg microsomal protein

^h^pmol/min/pmol CYP

^i^ISEF calculated on the basis of Eq. 4

In the next step, the CYP-specific ISEFs were defined. The ISEFs were calculated based on the measured apparent *V*max in incubations with Supersomes^™^ and pooled HLMs and are presented in Table 4. The highest ISEF value was found for CYP2B6 (0.48) and the lowest one for CYP1A2 (0.07).

**Fig. 4:**
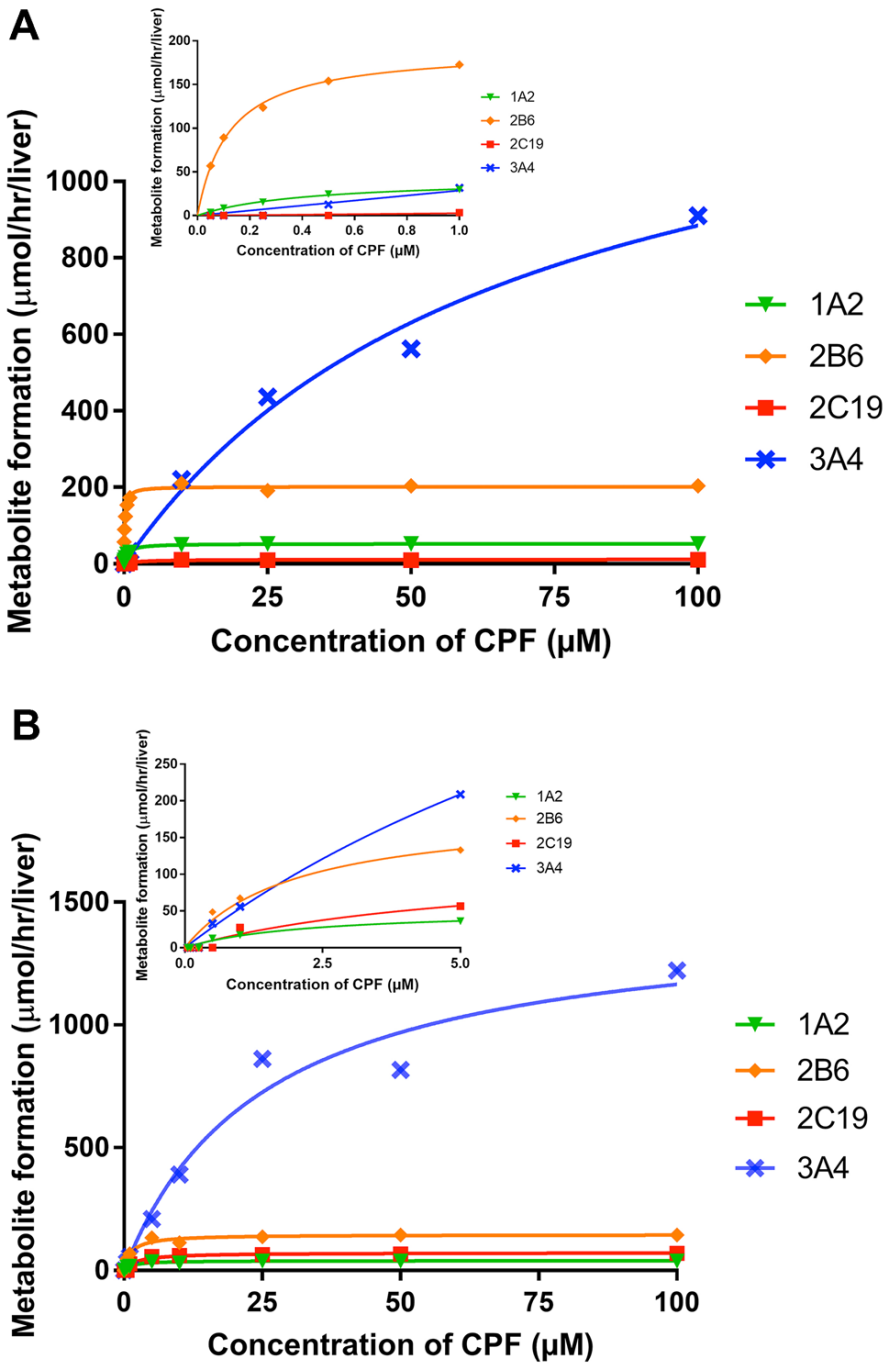
Concentration-dependent metabolic velocity of each CYP in whole liver for (A) bioactivation of CPF to CPO and (B) detoxification of CPF to TCPy. Since different concentration ranges were used in the different CYP incubations, the velocity of concentrations exceeding the incubation concentration range of each CYP were set equal to its corresponding Vmax value, to facilitate the graphical comparison. The insert presents the data at the lower concentration range (up to 1 µM in **A**, and 5 µM in **B**) in some more detail

To gain a better insight in the contribution of each CYP to the metabolism of CPF in vivo at different concentrations of CPF, the apparent Supersome^™^ kinetic data were scaled toward the whole liver according to Eq. 3. The corresponding Michaelis–Menten plot for each CYP upon extrapolation is presented in Fig. 4.

Figure 4A shows that at a low concentration (< 1 µM), CYP1A2 and especially CYP 2B6 play the primary role in bioactivation of CPF to CPO. However, with increasing concentration, CYP1A2 and CYP2B6 approach saturation while the 3A4-mediated conversion starts to become an important contributor to CPO formation from concentration levels higher than 10 µM onwards. CYP2C19 appears to have only a minor contribution at both low and high concentrations. These findings indicate that there are high affinity components (low Km for CYP1A2, 2B6 and 2C19) and a low affinity component (high Km for CYP 3A4) to be taken into account to adequately describe the bioactivation of CPF, resulting in biphasic kinetics. The one to two orders of magnitude lower Km for CYP1A2, 2B6 and 2C19 compared to the Km for CYP3A4 (Table 4) reflects this biphasic behavior. Differently, in detoxification of CPF (Fig. 4B), CYP3A4 plays a main role in the formation of TCPy at low concentrations, and it is becoming even more pronounced at CPF concentrations higher than 1 µM, with CYP1A2, CYP2B6 and CYP2C19 being involved in the detoxification pathway to a more limited extent.

Thus, the Supersome^™^ data depicted in Fig. 4 indicate biphasic kinetics to occur in the CYP-mediated bioactivation pathway, which is supported by the Eadie–Hofstee plot (Supplementary material III) for the summed enzyme velocities of the individual CYPs for pathway 1. However, the CYP-mediated detoxification pathway of CPF does not show such distinctive biphasic behavior, in line with the fact that Fig. 4b reveals that for this reaction the conversion at low concntrations was not dominated by a high affinity CYP, resulting in a comparable (1.4-fold difference) sum of the scaled catalytic efficiency (CE) values of the four individual Supersomes^™^ in pathway 2 at low and high concentration ranges, allowing the kinetics for this reaction to be described in a monophasic way.

To evaluate the performance of the Supersomes^™^ together with the ISEF to predict the bioactivation of CPF, the sum of the scaled CE of the four individual Supersomes^™^ was compared to the CE obtained for the pooled HLMs (for both non-biphasic and biphasic scenarios). The comparison reveals that the summed scaled CE of Supersomes^™^ (CE = 1578 l/h) is in line with that of pooled HLM when biphasic kinetics was taken into account (CE = 3600 l/h), but is not comparable when the high affinity phase was excluded (CE = 41 l/h), indicating it is of critical importance to include the high affinity phase when characterizing a metabolic conversion that is catalyzed by two or more CYP isoforms with distinct affinities. Given these results, the non-biphasic HLM-based approach was excluded in later steps presented below because of its incomplete characterization of CPF bioactivation.

#### In vitro metabolic conversion of CPO to TCPy in plasma

The apparent and scaled *V*max, *K*m, and CE for detoxification of CPO in plasma as well as the determined total protein concentration of plasma samples of 25 individuals are presented in Supplementary material IV. In general, the difference between the highest and the lowest CE is around 2.6-fold. This difference is mainly caused by individuals 15 and 16, due to a 1.5-fold lower apparent *K*m in individual 15 and a twofold lower apparent *V*max in individual 16 compared to the mean *K*m (290 µM (Table 2)) and *V*max (1844 nmol/min/ml plasma (Table 2) or 320,937 µmol/h (data not shown)), respectively. The calculated CV of the Vmax amounted to 0.29, and the CV for the Km was 0.33 (Table 2). The results also show that the 25 individuals have relatively comparable total protein concentrations in plasma, showing an only 1.3-fold difference between the highest and the lowest value (Supplementary material IV).

### PBK model evaluation

**Fig. 5:**
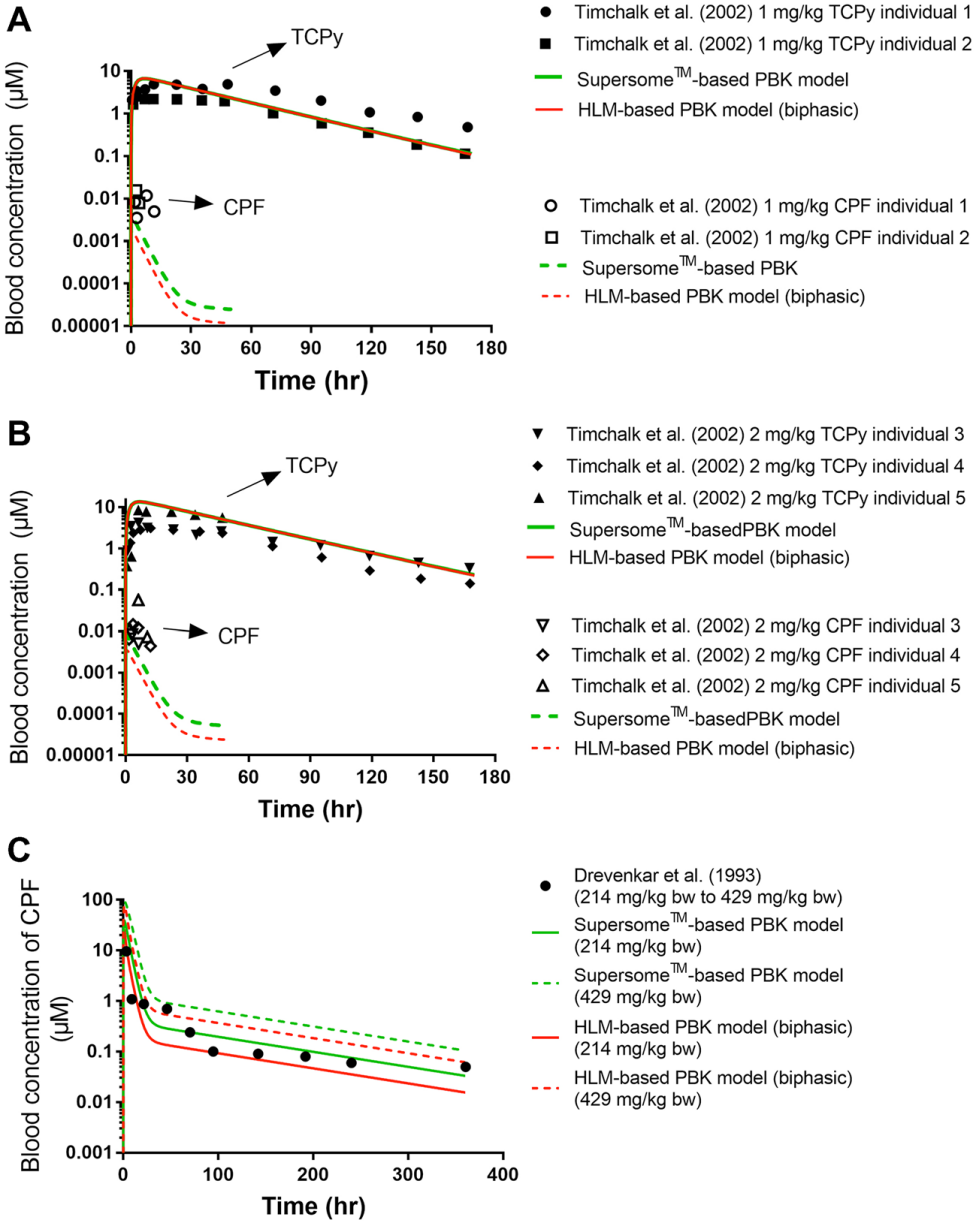
Comparison between reported in vivo data and PBK model predictions for time-dependent blood concentrations of CPF and time-dependent blood concentrations of TCPy at 1 mg/kg bw (**A**), and 2 mg/kg bw (**B**) (Timchalk et al. 2002), and 214 mg/kg bw (solid line) to 429 mg/kg bw (dash line) (**C**) the latter equal to the estimated dose range, for the estimated intake dose of poisoning victim A (Drevenkar et al. 1993)

The performances of the Supersome^™^ and HLM-based PBK models were evaluated against available in vivo data obtained from the literature (Fig. 5 and Supplementary material V). The data in the Supplementary material V reveal that the two models adequately predict the TCPy blood concentration, the predicted *C*_max_ value was 2.2-fold different from the mean value of *C*_max_ in in vivo data (Nolan et al. 1984) and the urinary TCPy excretion upon oral administration of 0.5 mg/kg bw is predicted to result in a concentration at 120 h that is 0.8-fold different from the in vivo value (Nolan et al. 1984). Similar results were observed when comparing the predicted *C*_max_ of TCPy from the two models with in vivo data from 5 individuals (Timchalk et al. 2002) (Fig. 5 A, and B), with the predictions being 1.3-fold to 4.0-fold different from the in vivo values. Apart from that, the two models were further evaluated by comparison of the predicted CPF blood concentration with the corresponding in vivo data from the 5 individuals. As illustrated in Fig. 5, an either 0.04- to 0.99-fold difference or 0.02- to 0.44-fold difference was found when comparing the predicted blood concentration of CPF by the Supersome^™^-based PBK model or the HLM-based PBK model, respectively to the reported blood concentration data. To better evaluate the time-dependent CPF concentration in blood, the predictions were also compared with CPF blood concentration data from Drevenkar et al. (1993) of a  poisoning victim. The results show that, at the estimated dose range of 214–429 mg/kg bw which reflects the estimated intake range of a poisoning victim, the CPF blood concentrations predicted by the Supersome^™^ and HLM-based PBK models are in line with the reported CPF blood concentration of a poisoning victim (Drevenkar et al. 1993).

### Sensitivity analysis

In the present study, the impact of each parameter of the Supersome^™^-based model and the HLM-based PBK model (biphasic) on the model output (free blood *C*_max_ of CPO) was determined by performing a sensitivity analysis. Only the parameters with normalized sensitivity coefficients higher than 0.1 (absolute value) are shown in Supplementary material VI. In general, no large difference was found in the sensitivity analysis of the two approaches. At low dose levels (0.5 mg/kg bw), the model output was mainly influenced by body weight, volume of liver, volume of blood, cardiac output, blood flow to liver and rapidly perfused tissue, ka, fa, liver microsomal protein yield scaling factor, the fuCPO _in vivo(plasma)_ as well as BPCPO. In addition to that, the free blood *C*_max_ of CPO appears also to be significantly affected by the kinetic parameters of CPO detoxification in both approaches, and to a lesser extent by CYP2B6-derived kinetic parameters of CPO bioactivation in the Supersome^™^-based model. At the exposure dose of 180 mg/kg bw, similar results were obtained, except that the parameters such as volume of liver, ka, fa, and liver microsomal protein yield scaling factor had less influence on the model output. In contrast, CYP-derived kinetic parameters of CPO bioactivation (especially CYP3A4) and its related parameters (ISEF and CYP abundance) in the Supersome^™^-based model, and HLM-derived kinetic parameters of CPO bioactivation in the HLM-based PBK model (biphasic) became influential on the model output.

### Monte Carlo simulation and CSAF (HK_AF_)

#### Including variability in metabolism-related kinetic parameters

The frequency distributions of the predicted *C*_max_ of CPO obtained using the Supersome^™^-based PBK model approach and the HLM-based PBK model approach including biphasic kinetics are presented in Supplementary material VII, and the corresponding GM, 95th and 99th percentile values of the distributions are shown in Table 5. Supplementary material VII and Table 5 together reveal that there are no substantial differences in the frequency distributions of the predicted *C*_max_ of CPO obtained by the two approaches, resulting in more or less similar GM, 95th and 99th percentile values between the two approaches (maximum 1.2-fold different). These values were used to calculate the HK_AF_ values, which were obtained by dividing the 95th or 99th percentile of the general population by the GM of the general population. As presented in Table 5, a slight difference was observed between both approaches when using the 95th percentile providing a HK_AF_ of 2.6 and 2.5 for the Supersome^™^-based PBK model and the HLM-based PBK model, respectively. When using the 99th percentile, the calculated HK_AF_ values were consistent, amounting to 3.6 for both approaches.

**Table 5:** Geometric mean values, the 95th and the 99th percentile values of the distribution for the predicted free blood maximum concentration (*C*max) of CPO after a single oral CPF dose of 0.47 mg/kg bw in Monte Carlo simulations, and its resulting CSAFs in the Supersome^™^-based PBK model and the HLM-based PBK model approach when taking only metabolism-related kinetic parameters into account or taking all influential parameters into account

	Including variability in metabolism-related kinetic parameters	
	Supersome^™^-based PBK model	HLM-based PBK model
CSAF(HK_AF_)^a^		
P95/GM	2.6	2.5
P99/GM	3.6	3.6
Total runs	58,792	9712

	Including variability in additional influential parameters	
	Supersome^™^-based PBK model	HLM-based PBK model
CSAF(HK_AF_)^a^		
P95/GM	4.1	4.1
P99/GM	6.9	6.6
Total runs	54,642	9013

^a^Chemical specific adjustment factor (CSAF) for human variability in toxicokinetics (HK_AF_)

#### Including variability in additional influential parameters

When taking the variation in other influential PBK model parameters into account in the Monte Carlo simulations, a 1.5–1.6 fold higher HK_AF_ value was obtained than that obtained when including only the variation of metabolism-related kinetic parameters, amounting to 4.1 for both approaches when using the 95th percentile (Table 5). At the 99th percentile, the HK_AF_ was affected to a somewhat larger extent, increasing 1.8–1.9 fold, being 6.9 for the Supersome^™^-based approach and 6.6 for the HLM-based approach (Table 5). Their corresponding frequency distributions are presented in Supplementary material VII.

### Reverse dosimetry to extrapolate in vitro AChE inhibition data to in vivo dose–response curves

**Fig. 6:**
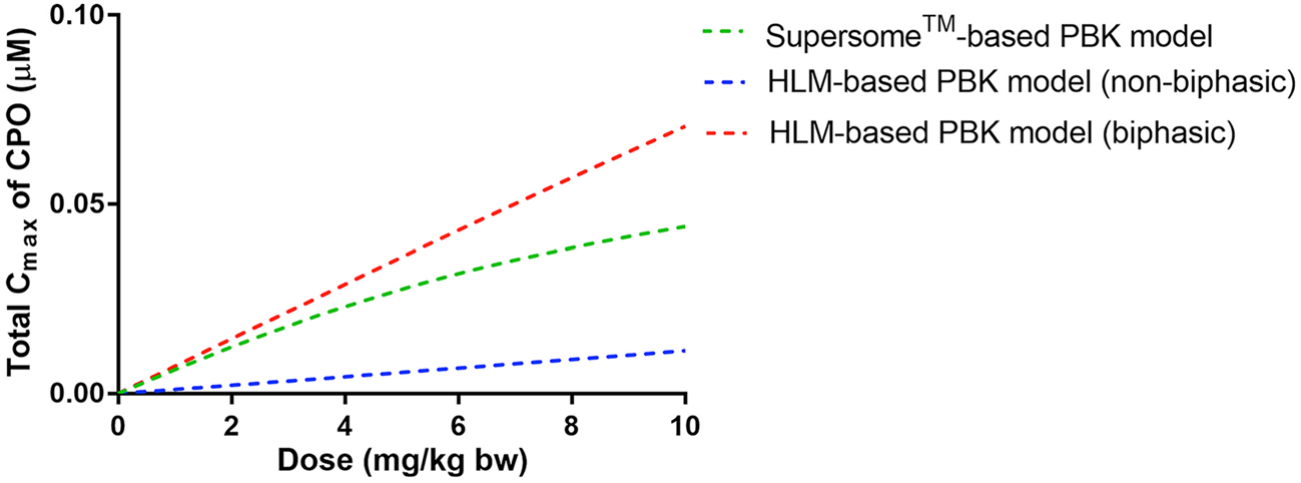
Comparison of the prediction for the CPF dose-dependent total *C*_max_ of CPO by the different approaches

For further evaluation of the newly defined PBK models, Fig. 6 shows a comparison of the CPF dose-dependent predicted total blood *C*_max_ of CPO using the PBK models developed in the present study. The comparison reveals that the predictions by especially the non-biphasic HLM-based model deviates from the predictions by the other models, while the data provided by the Supersome^™^-based PBK model and those obtained with the HLM-based PBK model (biphasic) were found to be similar, with a difference of 1.1-fold to 1.6-fold, increasing with the dose.

**Fig. 7:**
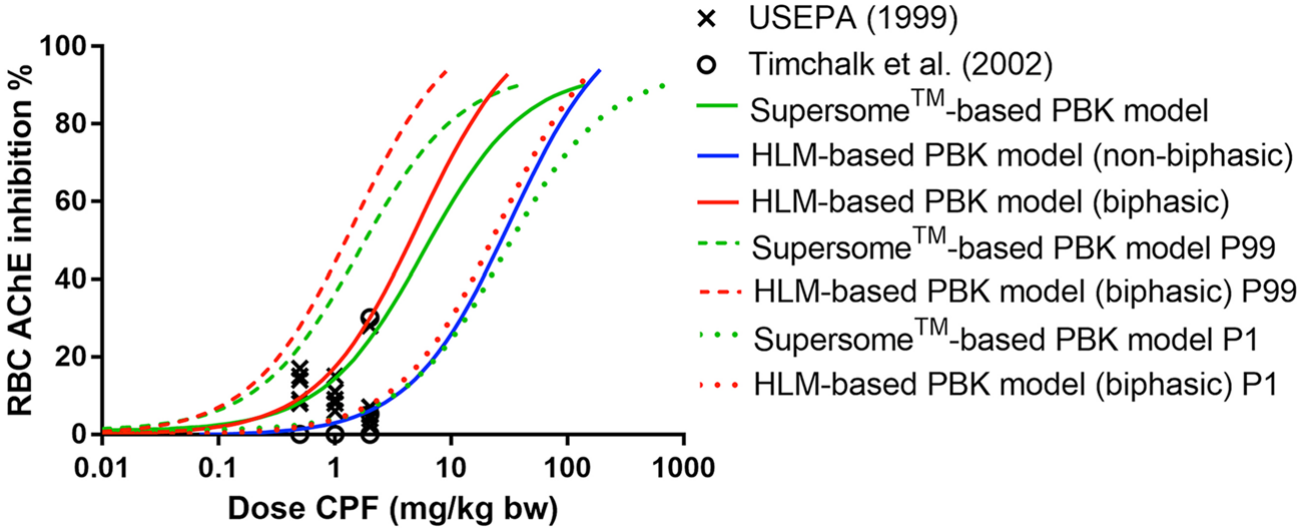
The predicted in vivo dose–response curves for AChE inhibition upon CPF exposure in human using the Supersome^™^-based PBK model (green solid line for average population, green dash line for 99th percentile sensitive individuals, and green dot line for 1st percentile sensitive individuals), the HLM-based PBK model (non-biphasic, blue solid line) and the HLM-based PBK model (biphasic, red solid line for average population, red dash line for 99th percentile sensitive individuals, and red dot line for 1st percentile sensitive individuals) for the reverse dosimetry. The individual data points represent available in vivo data for RBC AChE inhibition in human upon oral exposure to CPF at different dose levels as reported by USEPA (1999) and Timchalk et al. (2002) (color figure online)

Finally Fig. 7 illustrates that when using the different PBK models to translate the in vitro concentration–response curve for CPO-mediated inhibition of rhAChE inhibition to in vivo dose–response curves using reverse dosimetry, the predicted dose–response curves for the average population by the Supersome^™^-based PBK model and HLM-based PBK model (biphasic) were in line with in vivo data points, while the curve obtained with the HLM-based PBK model (non-biphasic) appeared to inadequately describe the actual inhibition reported at low dose levels for some individuals. Additionally, Fig. 7 presents the dose–response curves predicted for the 99th percentile and 1st percentile of the population, reflecting the possible inter-individual variation in the RBC AChE response predicted by the Supersome^™^-based PBK model approach and the HLM-based PBK model approach (biphasic). The curves obtained for 99th percentile and 1st percentile individuals by the Supersome^™^-based PBK model are similar to those predicted by the HLM-based PBK model (biphasic), except for the fact that the curves obtained with the HLM-based biphasic approach are somewhat steeper than those obtained with the Supersome^™^-based approach.

### BMD analysis and evaluation of the PBK model-based reverse dosimetry predictions

**Fig. 8:**
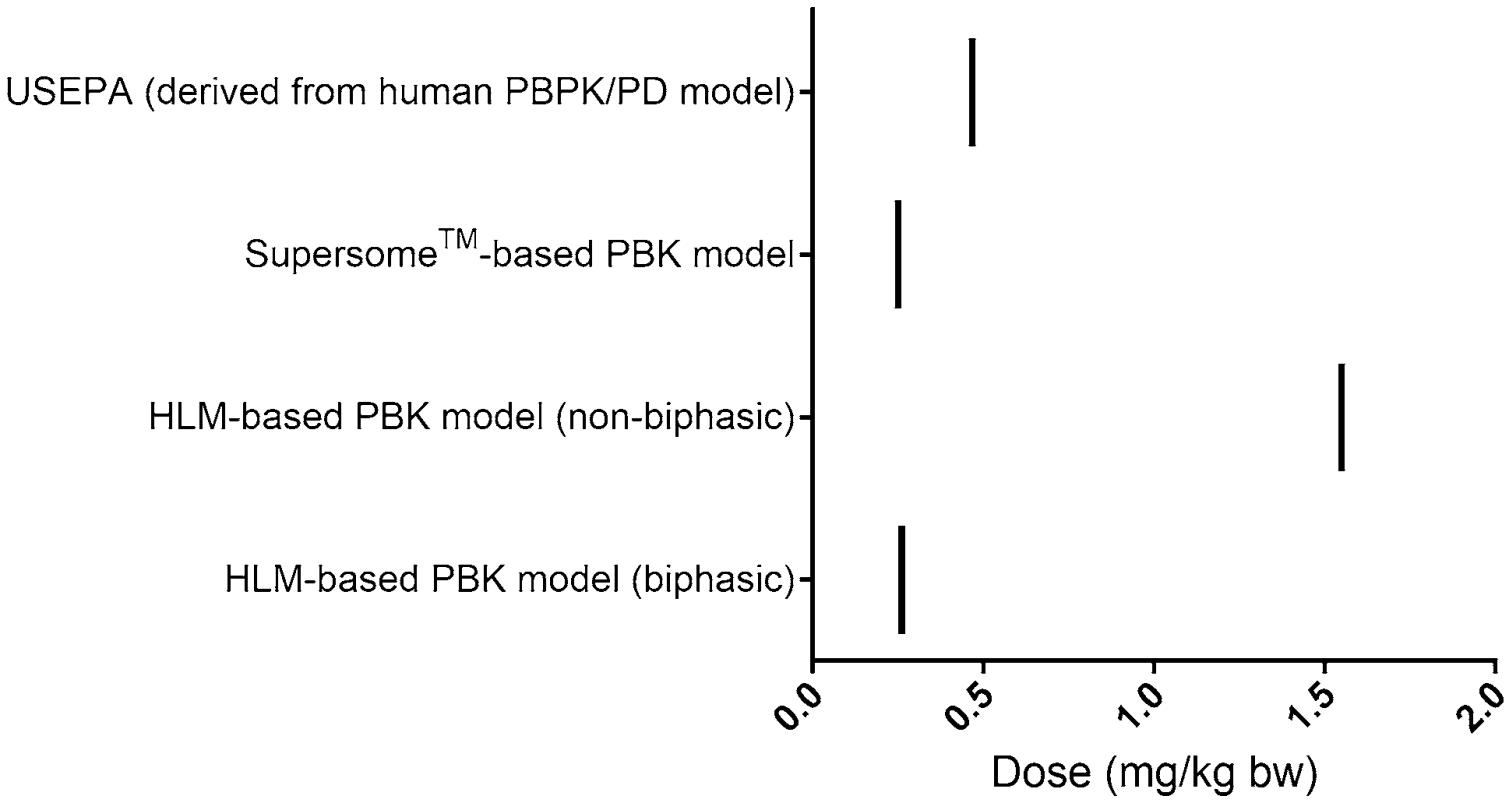
Comparison of predicted BMDL_10_ values by the present study to reported BMDL_10_ values established by USEPA (2014)

Figure 8 and Supplementary material VIII, present the BMDL_10_ values derived as PODs from the dose–response curves for the average population presented in Fig. 7. From these results it can be derived that the POD values obtained with the PBK model defined in the present study by the Supersome^™^-based PBK model and the HLM-based biphasic PBK model for the average population were comparable to the reported human model-derived BMDL_10_ values (1.9-fold and 1.8-fold lower, respectively) (USEPA 2014). However, when the high affinity component was excluded in the non-biphasic HLM-based PBK model, the predicted BMDL_10_ values was found to be 3.3-fold higher than the reported human model-derived BMDL_10_ values (USEPA 2014).

## Discussion

The present study compared the performance of two different approaches on characterization of the inter-individual variation in metabolism of CPF and its resulting RBC AChE inhibition using PBK modeling-based reverse dosimetry linked with Monte Carlo simulations. In one approach, variation in metabolism was calculated using Supersomes^™^ together with reported variation in CYP abundance, while in the other approach, variation was quantified using individual HLM data from Smith et al. (2011). The obtained results revealed that both approaches adequately predicted the time-dependent blood concentration profile of CPF and its metabolite TCPy, and could be used to successfully translate the in vitro concentration–response curves for RBC AChE inhibition by the active metabolite CPO to corresponding in vivo dose–response curves for CPF induced inhibition of RBC AChE activity, resulting in a derived BMDL_10_ value that was comparable to the reported BMDL_10_ value from the USEPA (2014). Additionally, the similar results obtained from the Supersome^™^-based approach and the HLM-based approach (biphasic) imply that there is a good match between the two approaches, thus also resulting in comparable HK_AF_ values. When comparing the obtained HK_AF_ values with the default uncertainty factor of 3.16, the HK_AF_ value derived using the 95th percentile is found to be well covered by the default value of 3.16, while the HK_AF_ defined using the 99th percentile, was slightly higher than 3.16, reflecting a possible inadequate protection by the default safety factor for a small part of the population. When also including the variability in other influential parameters in the Monte Carlo simulations, 1.5–1.6 or 1.8–1.9 fold higher HK_AF_ values were obtained at the 95^th^ and 99^th^ percentile respectively, reflecting to a further extent a possible inadequate protection by the default safety factor for a small part of the population.

In the present study, the IC50 value obtained from the CPO-induced concentration–response curve in rhAChE was found to be comparable to the IC50 value of 3.1 nM (taking into account a correction for the unbound fraction of CPF in plasma amounting to 0.15 (Heilmair et al. 2008)) reported in the study of Eyer et al. (2009). In this study the plasma samples from the patients poisoned with CPF were incubated with uninhibited RBCs from a healthy donor. This similar IC50 value indicates that rhAChE and native human RBC have a comparable sensitivity towards in vitro inhibition following CPO exposure.

The kinetic parameters obtained by Supersome^™^ incubations revealed that the bioactivation of CPF exhibits biphasic kinetics, based on the fact that the Km of CYP1A2, CYP2B6 and CYP2C19 were one to two orders of magnitude lower than that of CYP3A4 (Foxenberg et al. 2007). As a result, the combined kinetic data from these four main CYPs generated a characteristic pattern of biphasic kinetics in the Eadie-Hofstee plot (Supplementary material III). However, in incubations with HLMs, only the low affinity component was identified when using CPF at a concentration range of 3–100 µM (Sams et al. 2004; Zhao et al. 2019), while the high affinity component was not. These differences indicate that identifying such multiple CYP isoform-mediated biotransformation reactions of CPF using HLMs might be problematic. Different from the Supersome^™^-derived kinetic data that reflect each CYP-specific intrinsic kinetic profile and parameters (*K*_m_ and *V*_max_) towards the chemical of interest, the HLM-derived kinetic data represent the overall metabolic conversion by all relevant CYPs. Therefore, using substrate concentration ranges that far exceed the Km of some of the CYPs involved could lead to an incomplete capture of the kinetic phase, resulting in an under-estimation of the metabolic rate especially at low concentrations and dose levels. In theory, this issue can be overcome by extending the substrate incubation concentration range. In practice, however, the quantification of formation of CPO in incubations with HLMs at low substrate concentrations (in this case CPF < 1 µM) may be challenging, because of the detection limits for the metabolite of interest. Buratti et al. (2003) employed a highly sensitive detection method for CPO (based on AChE inhibition) for the metabolic reaction of CPF using HLMs. However, the assay was only able to identify the high affinity CYP kinetic components due to the complete inhibition of the AChE at high substrate concentrations and high formed CPO metabolite concentrations (Buratti et al. 2003). These observed incomplete captures of HLM kinetic data will occur less often with Supersome^™^, since they have a higher catalytic activity compared with the native CYPs present in HLM, allowing identification of both low and high affinity components using normal enzyme incubation assay conditions and established LC–MS/MS or UPLC-PDA detection methods.

Although Supersome^™^ seems a promising system for studying the metabolic turnover of chemicals, the activities of CYP enzymes from Supersome^™^ are different from native sources, as Supersome^™^ is a recombinant enzyme system made using baculovirus-transfected insect cells and exhibiting very high levels of catalytic activities. Therefore use of Supersome^™^ requires an ISEF to correct for differeces in activity compared to the HLMs, before scaling the activities to the in vivo situation. Applying such an ISEF may lead to an over- or under-estimation of metabolism, due to the possible variations in determination of ISEF values such as variability in the commercially supplied Supersome^™^ and HLM systems used, the probe substrate used, and the laboratory conditions applied (Chen et al. 2011; Proctor et al. 2004). In the present study the variabilities and inconsistencies related to ISEF values were minimized by using a probe substrate for the relevant CYPs in both enzyme systems to derive the ISEFs (instead of using default ISEF values), and using the same batch of Supersomes^™^ and HLMs for all ISEF determinations in the same laboratory. The overall extrapolated Vmax values using ISEF values established in the present study from Supersome^™^ kinetic data were found to be 1.9- and 0.9- fold different from the Vmax values derived from the HLM data for the high and low affinity component, repectively (Buratti et al. 2003; Sams et al. 2004; Smith et al. 2011; Zhao et al. 2019), indicating the adequency of the ISEF values established in the present study.

Different from the bioactivation of CPF to CPO (pathway 1), the detoxification of CPF (pathway 2) was shown in the present study to be adequately described by only one set of Vmax and Km. However, it is true that both bioactivation and detoxification of CPF were mainly catalyzed by similar type of CYPs, therefore biphasic kinetics may in theory also be expected to occur in the detoxification. Based on the results obtained, however, it appeared that the detoxification reaction kinetics were dominated by the contribution of only CYP3A4 at both low and high concentrations. Therefore, it eliminates the need for description of biphasic kinetics in pathway 2. A similar kinetic behavior for the conversion of CPF to TCPy was also observed in a previous study in rat liver microsomes (Ma and Chambers 1995).

The consequences of using different in vitro kinetic data as input of model-based reverse dosimetry were described by the BMDL_10_ values obtained in the present study. The BMDL_10_ value (for the average population) obtained using the HLM-based approach taking biphasic kinetics into account was found to be comparable to the BMDL_10_ values reported by the USEPA (2014), but was sixfold lower than that obtained from the HLM-based approach in which the high affinity component was not included. Such a difference is obviously due to the fact that without including the high affinity component, the contribution of CYP1A2, CYP2B6 and CYP2C19 at low concentrations to the bioactivation of CPF to CPO was neglected. Therefore, it is of importance to take biphasic kinetics into account, as an incomplete kinetic profile of bioactivation of CPF to CPO apparently results in an under-estimation of the POD leading to an inadequate protection of human health at especially realistic low dose exposure levels. When comparing the BMDL_10_ value obtained using the HLM-based PBK model that takes biphasic kinetics into account with the BMDL_10_ obtained with the Supersome^™^-based PBK model for the average population, no substantial difference was observed (1.04-fold), consistent with the comparable in vitro kinetic data predicted by the two approaches.

In the present study, for the Monte Carlo simulations, no covariation was assumed between different pathways. This was dependent on the following considerations. Based on the previous reported CE data for each pathway from Smith et al. (2011), the Pearson *r* was calculated (The correlation analysis, GraphPad Prism 5, version 5.04), which showed only low to mediate correlations between pathway 1 with either pathway 2 or 3 (Pearson *r* of Pathway 1 versus Pathway 2 = 0.63, and Pearson *r* of Pathway 1 versus Pathway 3 = 0.35), and mediate correlations between pathway 2 with pathway 3 (Pearson *r* = 0.51). Based on the fact that a low to moderate correlation will not substantially affect the model outcome (Bukowski et al. 1995; Poet et al. 2017), no correlations between these three pathways were included in the present study. Regarding Pathway 3 and 4 (PON1-mediated detoxification of CPO in liver and plasma, respectively), one may expect they will correlate with each other since plasma PON1 is known to be released from the liver (Ali and Chia 2008). However, there are no data that describe such a correlation between these two pathways. Moreover, by assuming that pathway 3 and pathway 4 completely correlate with each other, some possible combinations of kinetic data of the two pathways might be excluded in the Monte Carlo analysis. Therefore, to be conservative, these two pathways were treated as independent. Altogether, including no covariation between the different pathways maximizes the variability of the predicted CPO *C*_max_ (model output). As a result, the derived HK_AF_ values can be regarded to represent a conservative scenario.

Altogether, both the Supersome^™^-based model and the HLM-based model with biphasic kinetics appeared suitable to predict inter-individual variation in the metabolism of CPF, and this approach can be further advanced by considering the following improvement. In the present study, the HK_AF_ obtained relates to the general adult population, but did not yet relate to specific sub-population groups (e.g. pregnant women, infants and children). Some data suggest that there is a different capacity for CPO detoxification between specific sub-groups and general adults. Ferré et al. (2006) for example reported that the metabolic capacity can be altered during pregnancy, and that pregnancy can lead to an approximately 30% reduction in PON1 activity, which may result in a decreased capacity for CPO detoxification. In addition, an age-dependent increase of PON1 levels and activity have been reported (Huen et al. 2010), suggesting that human fetuses, infants, and young children may have lower capacity to detoxify CPO than adults. This observation has been corroborated by the human plasma kinetic data from Smith et al. (2011) reporting that Vmax values (on a per ml plasma basis) of CPO detoxification positively correlated with age. Therefore, the HK_AF_ derived in the present study may not provide full protection for these sensitive sub-groups. In further research, the Supersome^™^-based approach could be extended to also include sub-populations, allowing definition of a more comprehensive HK_AF_.

In conclusion, the present study demonstrates that using the Supersome^™^-based model and HLM-based model (biphasic), together with Monte Carlo simulations and reverse dosimetry can adequately predict the inter-individual variation in metabolism of CPF and its resulting RBC AChE response, providing comparable BMDL_10_ and HK_AF_ values in both approaches. Using Supersome^™^-derived kinetic parameters together with corresponding CYP abundances to describe inter-individual variability of CPF toxicokinetics enables to capture different kinetic affinity components, which may have been problematic when using HLM alone. On the other hand, using the Supersome^™^-based approach might be hindered by the accuracy of ISEF values used. Overall, taking these advantages and disadvantages of the two approaches into account, the Supersome^™^-based approach seems more appropriate than the HLM-based approach for identifying inter-individual variation in biotransformation of CPF and its resulting RBC AChE inhibition.

## Supplementary Information

Below is the link to the electronic supplementary material.

Supplementary file1 (PDF 553 KB)
