# Supplementary material for: Inter-individual variation in chlorpyrifos toxicokinetics characterized by physiologically based kinetic (PBK) and Monte Carlo simulation comparing human liver microsome and Supersome™ cytochromes P450 (CYP)-specific kinetic data as model input
Source: Arch Toxicol. 2022 Mar 16;96(5):1387–409. doi: 10.1007/s00204-022-03251-z (PMC9013686; doi:10.1007/s00204-022-03251-z)
Supplement: Supplementary file 1 — Supplementary file1 (PDF 553 KB) [file 204_2022_3251_MOESM1_ESM.pdf]

Inter-individual variation in chlorpyrifos toxicokinetics characterized by physiologically based kinetic (PBK) and Monte Carlo simulation comparing human liver microsome and Supersome^{TM} cytochromes P450 (CYP)-specific kinetic data as model input

Shensheng Zhao[a,1], Sebastiaan Wesseling[a], Ivonne. M.C.M. Rietjens[a], Marije Strikwold[b]

[a] Division of Toxicology, Wageningen University and Research, Stippeneng 4, 6708 WE, Wageningen, The Netherlands

[b] Van Hall Larenstein University of Applied Sciences, 8901 BV Leeuwarden, The Netherlands

[1]Corresponding author:

Shensheng Zhao

Division of Toxicology, Wageningen University and Research

Stippeneng 4, 6708 WE Wageningen

The Netherlands

E-mail: shensheng.zhao@wur.nl

Supplementary material I

Ultra-performance liquid chromatography-Photodiode Array (UPLC-PDA)

The amounts of the parent compound chlorpyrifos (CPF) and formed 3,5,6-trichloro-2-pyridinol (TCPy) in samples from the Supersome™ CYP and human plasma (HP) incubations were identified and quantified using a Shimadzu Nexera X2 LC-30AD UPLC coupled with a Shimadzu Photodiode Array Detector SPD-M30A (UPLC-PDA, Kyoto, Japan). The chromatographic separations were conducted on a Waters Acquity UPLC BEH C18 column (1.7 µm, 2.1 x 50 mm). The injection volume was 20 µl for the Supersome™ CYP samples and 3.5 µl for the plasma samples. The flow rate was 0.3 ml/min, and the temperature of the column was kept at 40ºC. The mobile phases used for the analysis consisted of (A) 0.1% trifluoroacetic acid (TFA) in nanopure water and (B) 100% acetonitrile (ACN). For identification and quantification of the parent compound CPF and formed TCPy, the gradient started with 10% B and was linearly increased to 100% B in 6.00 min, kept at 100% B for 50 sec and then changed to 0% B in 10 sec and maintained for 1 min before changing back to the initial condition (10% B) at 8.10 min and kept for 3.20 min to re-equilibrate the column before the next injection. With these conditions, the retention times of CPF and TCPy were 6.60 and 4.37 min, respectively. The amounts of CPF and TCPy were quantified by integrating the peak areas at 299 nm using calibration curves that were prepared using commercially available standards.

The identification and quantification of the probe substrates bupropion, phenacetin, (S)-mephenytoin, testosterone and their metabolites acetaminophen, 4-hydroxymephenytoin and 6β-hydroxytestosterone were performed using the same instrument and conditions as described above, except for the gradient. For identification and quantification of these probe substrates, the gradient started with 0% B and was linearly increased to 100% B in 6.00 min, kept at 100% B for 50 sec and then changed to the initial conditions (0% B) at 7.00 min and kept for 4.00 min to re-equilibrate the column before the next injection. The injection volume was 20 µl. With these conditions, the retention times of bupropion, phenacetin, (S)-mephenytoin, testosterone and metabolites acetaminophen, 4-hydroxymephenytoin and 6β-hydroxytestosterone were 4.06, 4.00, 4.28, 4.95, 3.00, 3.70 and 4.00 min, respectively. The amount of phenacetin, (S)-mephenytoin, testosterone and their metabolites acetaminophen, 4-hydroxymephenytoin and 6β-hydroxytestosterone was quantified at 240 nm, and bupropion was quantified at 210 nm, based on their corresponding calibration curves that were prepared using commercially available standards.

Liquid Chromatography Mass Spectrometry (LC-MS/MS)

The amounts of CPO formed in samples from the Supersome^{TM} CYP incubations were identified and quantified using a Shimadzu Nexera LC-40D$_{XR}$ Ultra-High-Performance Liquid Chromatography (UHPLC) system coupled to a Shimadzu LCMS-8045 mass spectrometer (Kyoto, Japan) equipped with an electrospray ionization (ESI) interface. The chromatographic separations were conducted on a Kinetex® Phenyl-Hexyl 100Å LC column (1.7 µm, 100 x 2.1 mm). The injection volume was 1 µl. A flow rate of 0.3 ml/min was applied and the temperature of the column was kept at 40ºC. The mobile phases used for the analysis consisted of (A) 0.1% (v/v) formic acid in ultrapure water and (B) 0.1% (v/v) formic acid in 100% ACN. The instrument was used in positive ionization mode with multiple reaction monitoring (MRM). For identification and quantification of CPO, the gradient started with 0% B and was linearly increased to 10% B in 1.00 min, and then to 40% B at 13.00 min, and continuously increased to 100% B at 18.00 min, kept at 100% B for 1.00 min and then linearly changed to the initial conditions (0% B) at 20.00 min and kept for 4.00 min to re-equilibrate the column before the next injection.

The identification and quantification of (±)-hydroxybupropion (metabolite of bupropion) was performed using the same instrument and conditions as described above, except for the injection volume that was 0.5 µl, and the gradient elution. For identification and quantification of (±)-hydroxybupropion, the gradient started at 0% B, changing to 60% B from 0.00 to 5.00 min, and linearly increasing further to 70% B at 9.00 min, and returning to the initial conditions at 10.00 min, at which it was maintained for 4.80 min to re-equilibrate the column before the next injection.

LC-MS/MS acquisition parameters

**Compound**	**Precursor ion (m/z)**	**Product ion (m/z)**	**Collision energy (V)**	**Retention time (min)**
chlopyrifos oxon (CPO)	333.95	277.90	-19	17.4
		198.05	-32	
		306.05	-12	
(±)-hydroxybupropion	256.00	238.10	-12	5.8
		167.10	-21	
		139.10	-26	

Supplementary material II

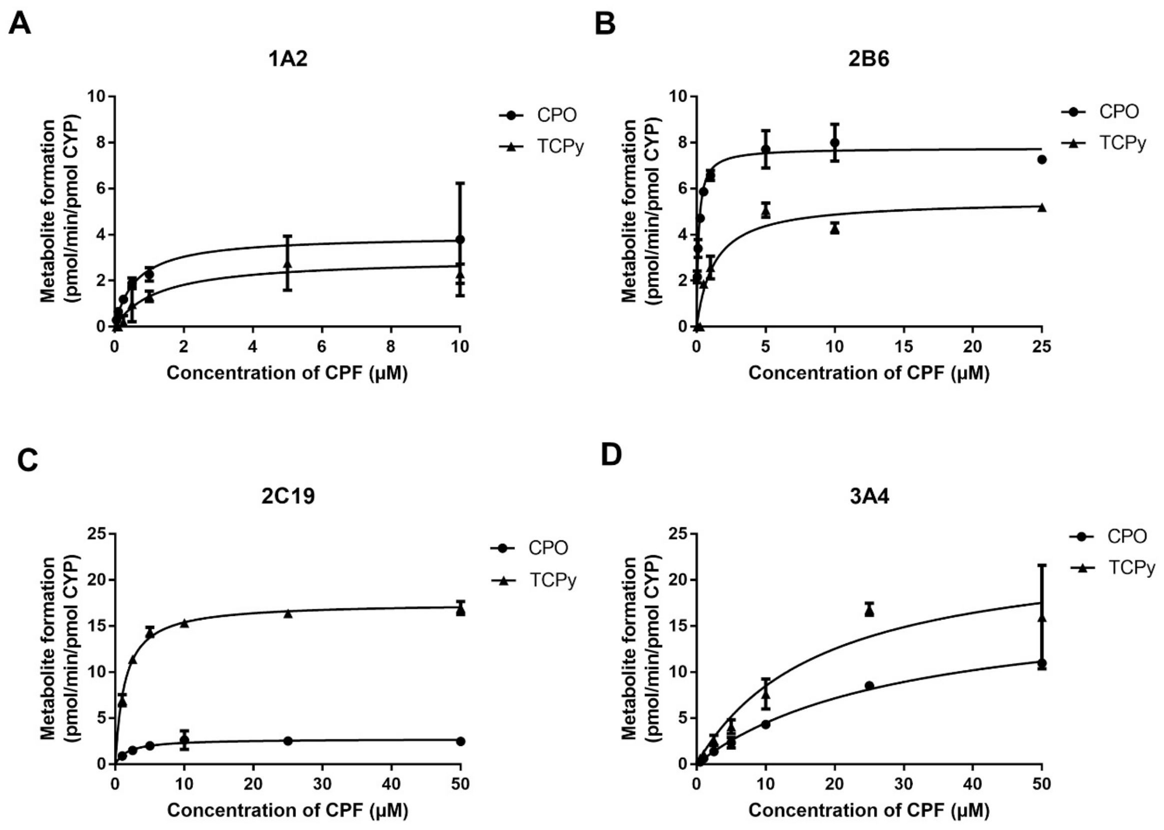

CYP450-mediated CPF concentration-dependent formation of TCPy (filled triangle) and CPO (filled circle) in incubations with human Supersome^{TM} CYP1A2 (A), CYP2B6 (B), CYP 2C19 (C) and CYP3A4 (D). Data points represent mean ± SD of two experiments for each conversion.

Supplementary material III

Eadie-Hofstee plot

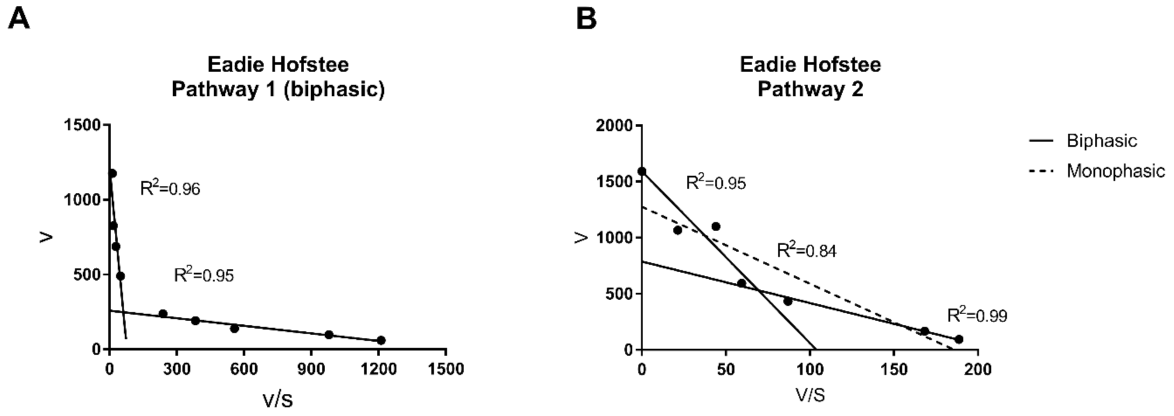

Eadie-Hofstee plot (Vmax against Vmax/CPF concentration (V against V/S)) for (A) pathway 1 (biphasic) and (B) pathway 2 (for both monophasic and biphasic kinetics).

Supplementary material IV

Kinetic parameters (Vmax and Km) and catalytic efficiency (CE) (calculated as Vmax/Km) for the detoxification of CPO to TCPy in incubations with plasma from 25 Caucasian individuals. Data represent mean of two experiments for each parameter.

Number	Gender	Age	Total protein [a]	$Km_{(app)}$ [b]	Vmax $_{(app)}$ [c]	Vmax [d]	CE [e]	Scaled Vmax [f]	Scaled CE [g]
1	Male	64	73.3	388.3	21.78	1596	4.1	291247	750.1
2	Male	65	71.2	310.4	17.32	1233	4.0	224952	724.7
3	Male	56	75.9	379.7	32.15	2439	6.4	445176	1172.4
4	Male	52	71.1	317.1	22.39	1591	5.0	290427	915.9
5	Male	51	72.4	637.6	45.36	3284	5.2	599306	939.9
6	Male	57	80.9	327.1	23.22	1878	5.7	342626	1047.5
7	Female	25	80.3	152.3	14.99	1203	7.9	219543	1441.5
8	Female	38	78.0	227.8	21.31	1663	7.3	303506	1332.3
9	Female	38	78.5	282.8	27.46	2157	7.6	393583	1391.7
10	Female	32	82.0	258.7	28.9	2370	9.2	43441	1671.6
11	Female	22	75.4	205.1	17.42	1314	6.4	239710	1168.7
12	Female	46	92.0	299.3	26.75	2461	8.2	449105	1500.5
13	Female	51	72.8	225.7	20.58	1498	6.6	273437	1211.5
14	Female	75	72.8	357.5	28.36	2064	5.8	376692	1053.7
15	Female	55	74.2	187.3	24.79	1840	9.8	335867	1793.2
16	Female	53	80.8	247.7	11.28	912	3.7	166366	671.6
17	Female	50	71.4	282	32.03	2287	8.1	417377	1480.1
18	Female	58	74.6	200.7	15.18	1132	5.6	206569	1029.2
19	Male	48	73.0	249.8	18.21	1329	5.3	242612	971.2
20	Male	49	84.9	307.6	24.85	2111	6.9	385226	1252.4
21	Male	44	79.6	283.4	23.05	1835	6.5	334943	1181.9
22	Male	34	71.8	237.6	24.05	1726	7.3	315041	1325.9
23	Male	47	89.0	373.9	24.05	2141	5.7	390628	1044.7
24	Male	28	82.9	269.7	28.44	2357	8.7	430169	1595.0
25	Male	41	75.7	236.5	22.13	1676	7.1	305884	1293.4

[a] mg/ml

[b] µM

[c] nmol/min/mg plasma protein

[d] Vmax (nmol/min/ml plasma), calculated by multiplying Vmax $_{(app)}$ (nmol/min/mg plasma protein) with total protein content (mg/ml)

[e] CE = catalytic efficiency (ml/min/ml plasma), calculated as Vmax (nmol/min/ml plasma) /$Km_{(app)}$ (uM)

[f] Scaled Vmax (µmol/hr), calculated based on Eq 7

[g] Scaled CE (l/hr), calculated as scaled Vmax (µmol/hr) /$Km_{(app)}$ (uM)

Supplementary material V

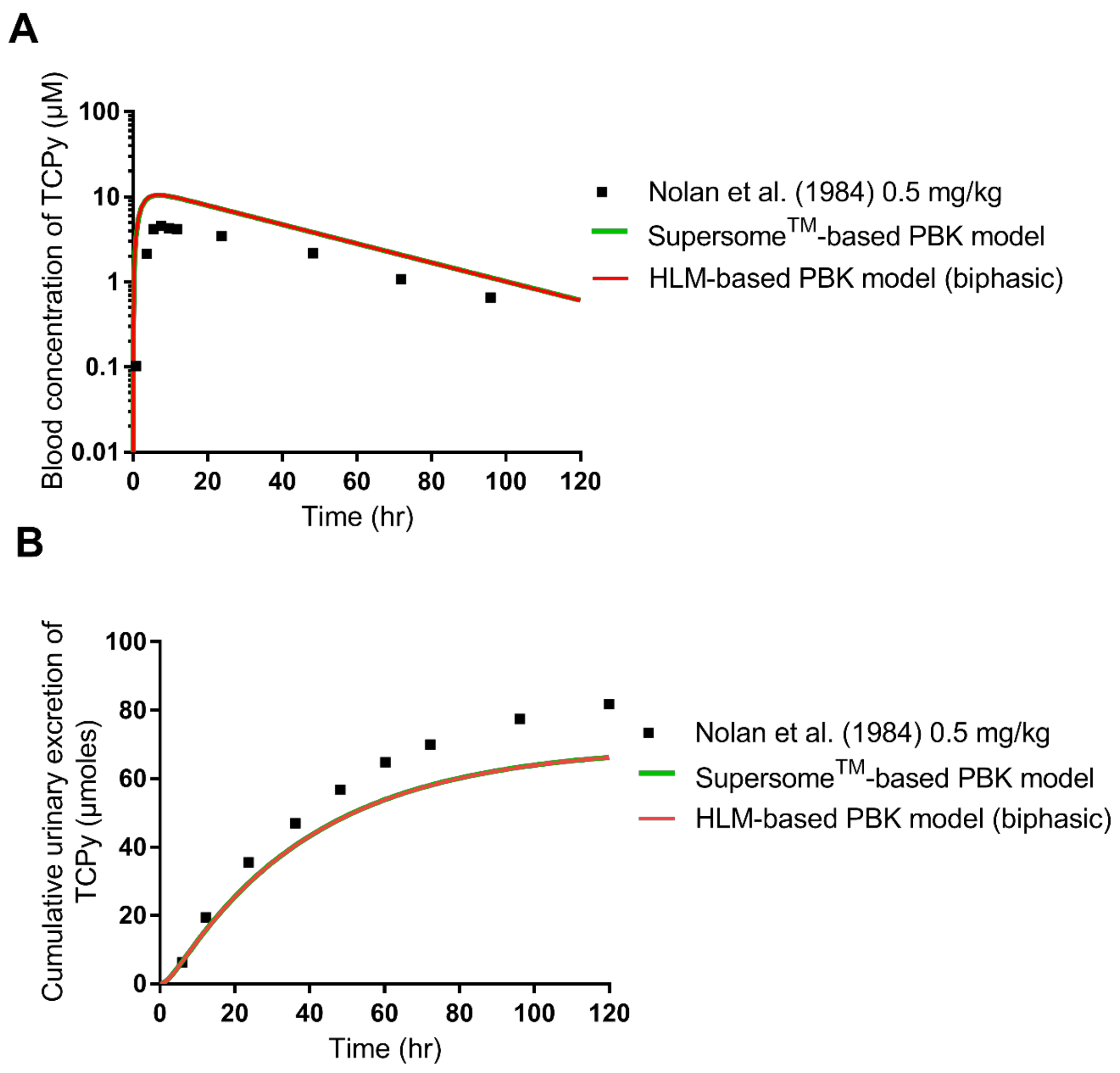

Comparison between reported in vivo data and PBK model predictions for (A) time-dependent blood concentrations of TCPy at a dose of 0.5 mg/kg bw (Nolan et al. 1984), (B) time-dependent urinary TCPy excretion at a dose of 0.5 mg/kg bw (Nolan et al. 1984)

Supplementary material VI

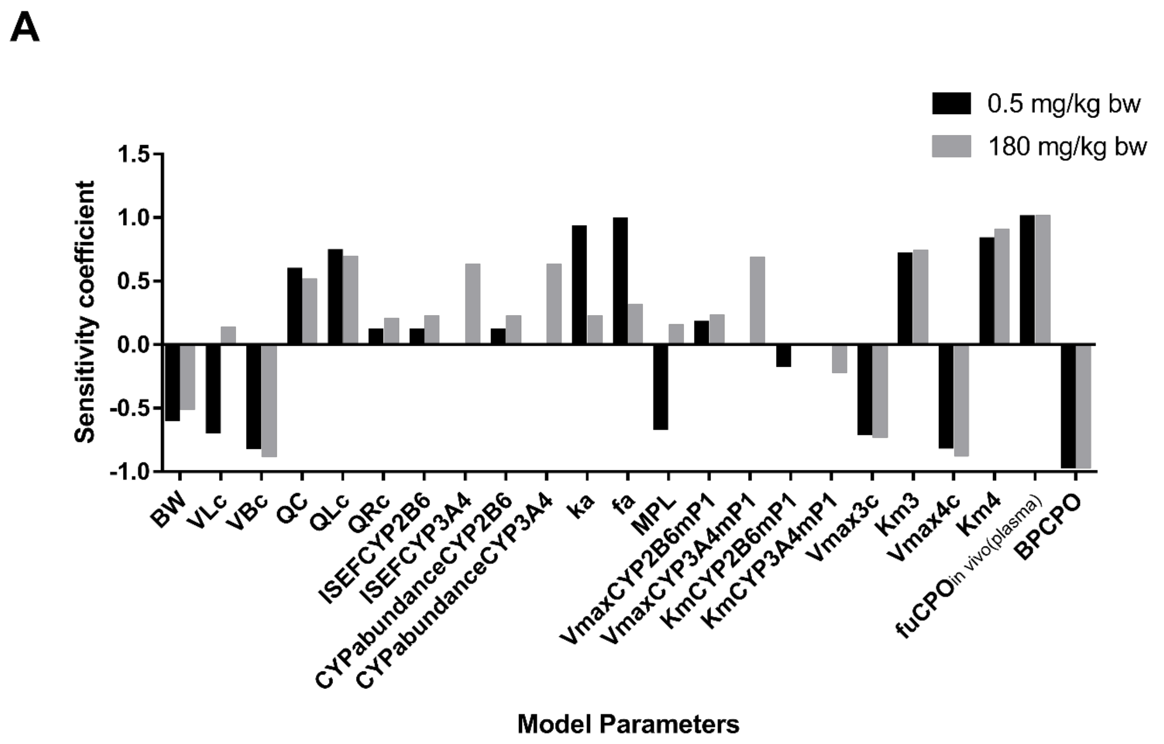

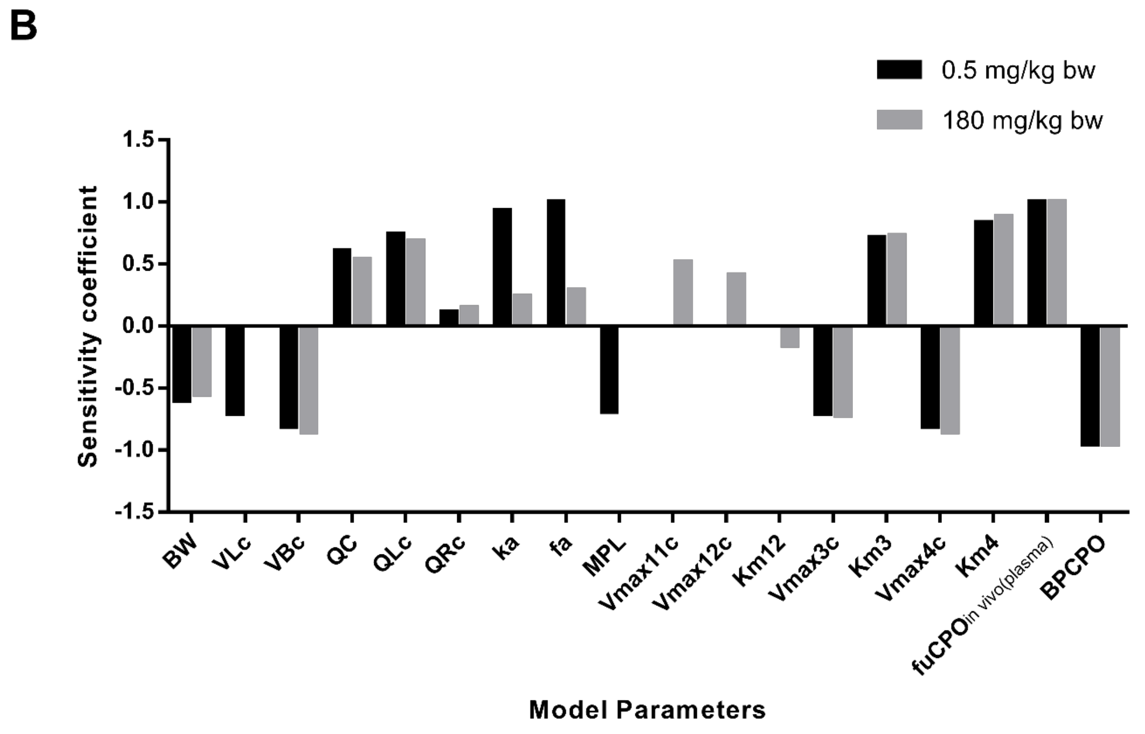

Sensitivity analysis for the predicted free blood C_{max} concentration of CPO at a low dose level of CPF of 0.5 mg/kg and a high dose level of 180 mg/kg bw by the Supersome^{TM} CYP-based PBK model (A) and the HLM-based PBK model (biphasic) (B). The parameters represent: BW body weight, VLc fraction of liver tissue, VBc fraction of blood, QC cardiac output, QLc fraction of blood flow to liver, QRc fraction of blood flow to richly perfused tissue,

ISEFCYP2B6 intersystem extrapolation factors for CYP2B6, ISEFCYP3A4 intersystem extrapolation factors for CYP3A4, CYPabundance2B6 average endogenous abundance (pmol CYP/mg microsomal protein) of CYP2B6 isoform in human liver microsome, CYPabundance3A4 average endogenous abundance (pmol CYP/mg microsomal) of CYP3A4 isoform in human liver microsome, ka first-order rate constant for absorption CPF from stomach into liver, fa fractional absorption, MPL liver microsomal protein yield, VmaxCYP2B6mP1 maximum rate for conversion of CPF to CPO by CYP2B6, VmaxCYP3A4mP1 maximum rate for conversion of CPF to CPO by CYP3A4, KmCYP2B6P1 Michaelis Menten constant for conversion of CPF to CPO by CYP2B6, KmCYP3A4P1 Michaelis Menten constant for conversion of CPF to CPO by CYP3A4, Vmax11c maximum rate for conversion of CPF to CPO at low concentration range by HLM, Vmax12c maximum rate for conversion of CPF to CPO at high concentration range by HLM, Km12 Michaelis Menten constant for conversion of CPF to CPO at high concentration range by HLM, Vmax3c maximum rate for conversion of CPO to TCPy by HLM, Km3 Michaelis Menten constant for conversion of CPO to TCPy by HLM, Vmax4c maximum rate for conversion of CPO to TCPy by HP, Km4 Michaelis Menten constant for conversion of CPO to TCPy by HP, fuCPO free fraction of CPO in vivo, BPCPO blood to plasma ratio of CPO.

Supplementary material VII

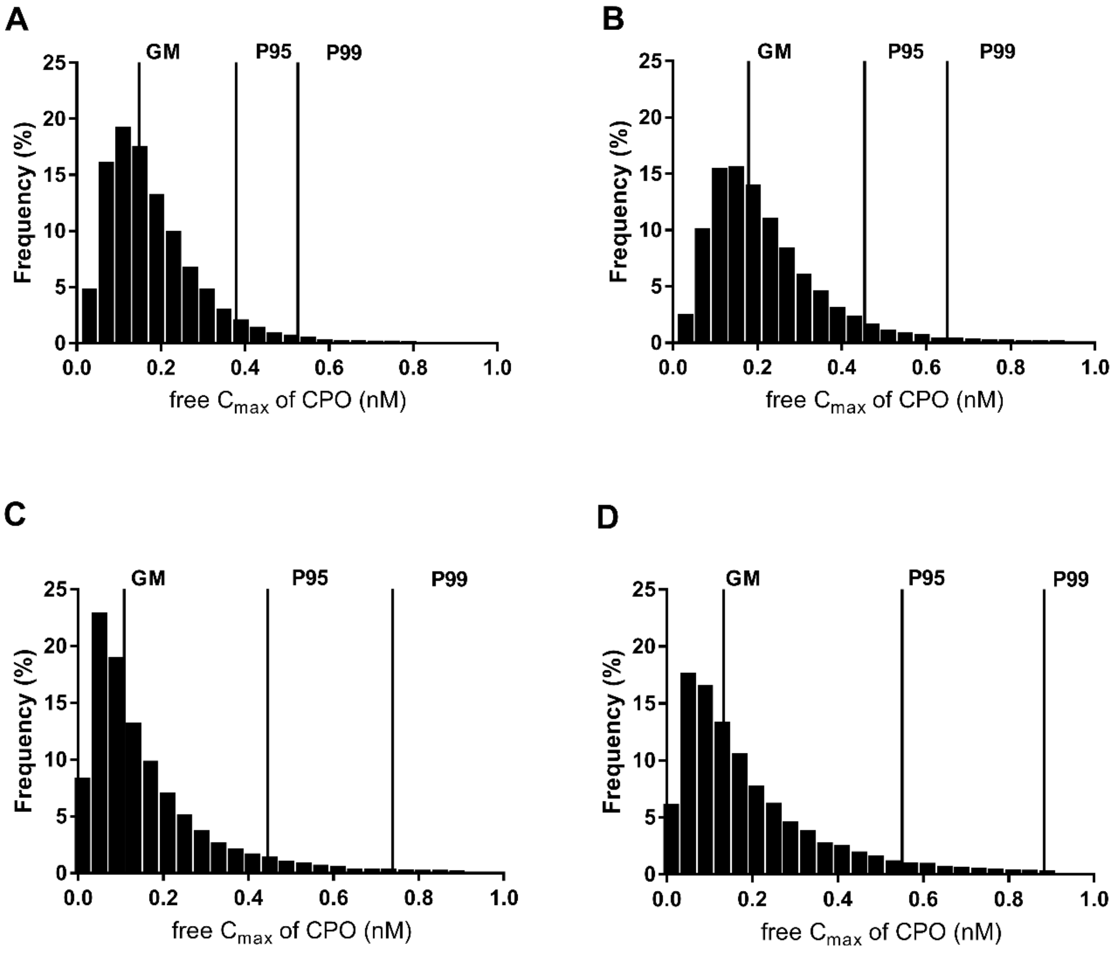

Frequency distribution for the predicted blood free maximum concentration (Cmax) of CPO after a single oral CPF dose of 0.47 mg/kg bw using the Supersome^{TM} CYP-based PBK model approach taking only the variation in metabolism-related kinetic parameters into account (A) or including also the variation of additional influential parameters (C) in the Monte Carlo simulations, and using the HLM-based PBK model approach (biphasic) taking only the variation in metabolism-related kinetic parameters into account (B) or including also the variation of additional influential parameters (D) in the Monte Carlo simulations, The GM, P95 and P99 represent the geometric mean, the 95th and the 99th percentile of the distribution.

Supplementary material VIII

Comparison of predicted $BMDL_{10}$ values from the present study to the reported $BMDL_{10}$ value established by the USEPA (2014)

	**Prediction**			**Reported**
	**Supersome^{TM} CYP-based approach**	**HLM-based approach (non-biphasic)**	**HLM-based approach (biphasic)**	**EPA $BMDL_{10}$ (derived from human PBPB-PD* model)**
**$BMDL_{10}$ (mg/kg bw)**	0.25	1.55	0.26	0.47

* Physiologically-based pharmacokinetic-pharmacodynamic (PBPK-PD) model
